# Metabolic Constraint-Based Refinement of Transcriptional Regulatory Networks

**DOI:** 10.1371/journal.pcbi.1003370

**Published:** 2013-12-05

**Authors:** Sriram Chandrasekaran, Nathan D. Price

**Affiliations:** 1 Institute for Systems Biology, Seattle, Washington, United States of America; 2 Center for Biophysics and Computational Biology, University of Illinois, Urbana-Champaign, Illinois, United States of America; Tel-Aviv University, Israel

## Abstract

There is a strong need for computational frameworks that integrate different biological processes and data-types to unravel cellular regulation. Current efforts to reconstruct transcriptional regulatory networks (TRNs) focus primarily on proximal data such as gene co-expression and transcription factor (TF) binding. While such approaches enable rapid reconstruction of TRNs, the overwhelming combinatorics of possible networks limits identification of mechanistic regulatory interactions. Utilizing growth phenotypes and systems-level constraints to inform regulatory network reconstruction is an unmet challenge. We present our approach *Gene Expression and Metabolism Integrated for Network Inference* (GEMINI) that links a compendium of candidate regulatory interactions with the metabolic network to predict their systems-level effect on growth phenotypes. We then compare predictions with experimental phenotype data to select phenotype-consistent regulatory interactions. GEMINI makes use of the observation that only a small fraction of regulatory network states are compatible with a viable metabolic network, and outputs a regulatory network that is simultaneously consistent with the input genome-scale metabolic network model, gene expression data, and TF knockout phenotypes. GEMINI preferentially recalls gold-standard interactions (p-value = 10^−172^), significantly better than using gene expression alone. We applied GEMINI to create an integrated metabolic-regulatory network model for *Saccharomyces cerevisiae* involving 25,000 regulatory interactions controlling 1597 metabolic reactions. The model quantitatively predicts TF knockout phenotypes in new conditions (p-value = 10^−14^) and revealed potential condition-specific regulatory mechanisms. Our results suggest that a metabolic constraint-based approach can be successfully used to help reconstruct TRNs from high-throughput data, and highlights the potential of using a biochemically-detailed mechanistic framework to integrate and reconcile inconsistencies across different data-types. The algorithm and associated data are available at https://sourceforge.net/projects/gemini-data/

## Author Summary

Cellular networks, such as metabolic and transcriptional regulatory networks (TRNs), do not operate independently but work together in unison to determine cellular phenotypes. Further, the phenotype and architecture of one network constrains the topology of other networks. Hence, it is critical to study network components and interactions in the context of the entire cell. Typically, efforts to reconstruct TRNs focus only on immediately proximal data such as gene co-expression and transcription factor (TF)-binding. Herein, we take a different strategy by linking candidate TRNs with the metabolic network to predict systems-level responses such as growth phenotypes of TF knockout strains, and compare predictions with experimental phenotype data to select amongst the candidate TRNs. Our approach goes beyond traditional data integration approaches for network inference and refinement by using a predictive network model (metabolism) to refine another network model (regulation) – thus providing an alternative avenue to this area of research. Understanding how the networks function together in a cell will pave the way for synthetic biology and has a wide-range of applications in biotechnology, drug discovery and diagnostics. Further we demonstrate how metabolic models can integrate and reconcile inconsistencies across different data-types.

## Introduction

The inference of transcriptional regulatory networks (TRNs) from high-throughput data is a central challenge in systems biology. TRN models provide a mechanistic framework for describing interactions between transcription factors and their target genes. Cellular phenotypes are influenced by the differential activity of these networks, and reconstructing the regulatory network enables one to understand the underlying molecular processes that cause phenotypic changes and better predict the response of a cell to an external perturbation.

Current network inference algorithms enable rapid reconstruction of TRNs by utilizing high-throughput data such as protein-DNA binding, DNA sequence or gene expression [1]–[12]. However, the overwhelming number of possible regulatory interactions between thousands of genes and transcriptional regulators in a cell—combined with the complex and dynamic nature of these interactions—limits the success of these inference approaches [1], [13], [14]. Recent analyses in *Saccharomyces cerevisiae* (baker's yeast) have shown that even though there are a multitude of predicted interactions, very few have a functional effect on the pathway activity or the metabolic flux distributions [13], [15]. Furthermore, a large-scale comparative study of expression-based network inference algorithms found poor performance in yeast [1]. One reason for this is that a connection to a growth or metabolic phenotype is missing during the inference process, making it difficult to assess the plausibility of the predicted interactions in a systems context. Connecting TRN inference to the phenotype data can lead to a more seamless connection between genomic measurements and phenotype.

We hypothesized that integrating regulatory interactions with metabolic networks would make it possible to more directly connect the regulatory interactions with their downstream phenotype, and thus allow us to use a broader range of data for network curation. Genome-scale models of metabolic networks have been constructed using growth phenotype data for a wide range of organisms, and these models accurately predict the response of the cell to environmental and genetic perturbations [16]–[19]. These models explicitly represent the mechanistic relationships between genes, proteins, and the chemical inter-conversion of metabolites within a biological system. The success of this integration would then allow the utilization of large-scale phenotypic data, which are commonly used to curate metabolic networks [16], [20], [21], to also refine regulatory interactions.

To enable the concurrent analysis of transcriptional regulation and metabolism, we recently developed the Probabilistic Regulation of Metabolism (PROM) approach for integrating biochemical networks with TRNs in an automated fashion [22]. We used PROM to demonstrate that phenotypic states can be predicted from the combined TRN and metabolic network models. PROM takes in a genome-scale metabolic network model, a regulatory network structure consisting of TFs and their targets, and gene expression data across different conditions, as inputs to predict the phenotypic outcome of transcriptional perturbations.

PROM solves the forward problem of combining disparate networks to predict phenotype (e.g., flux and growth rates). In the work described herein, we iteratively use PROM to aid in solving the more challenging inverse problem [23]—guiding TRN structure prediction using the metabolic network and the emergent phenotype measurements. In doing so, our new method serves as a tool to refine the inferred TRN and improve the predictive power of the integrated network models.

This new approach, Gene Expression and Metabolism Integrated for Network Inference (GEMINI), discerns functional regulatory interactions in high-throughput data by taking advantage of PROM, the growing amount of information in phenotype databases, and the observation by Barrett *et al* [24] that only a fraction of functional regulatory network states are compatible with a viable metabolic network. GEMINI produces a regulatory network state that is simultaneously consistent with observed gene knockout phenotypes, gene expression data, and the corresponding metabolic network state. While there have been approaches to model the constraints imposed by regulation and signaling networks on metabolism [18], [22], [25], [26] or to readjust manually curated regulatory rules based on metabolism [18], [27], no method thus far have utilized metabolic constraints to refine high-throughput interaction data as GEMINI does.

Here we describe the GEMINI approach and then test it by building a genome-scale integrated model for yeast. We compare the refined network model across various high-throughput data sets, and demonstrate that GEMINI effectively recalls known mechanistic interactions. We then iteratively expand and refine the integrated model using published genome-wide chromatin immunoprecipitation, TF knockout gene expression and binding-site-motif data sets, and show the ability of our integrated metabolic and regulatory network model to predict growth phenotypes of transcription factor knockout strains in new conditions. We also use GEMINI to identify potential condition-specific interactions and post-transcriptional regulatory mechanisms in *S. cerevisae*.

## Results

### Overview of the GEMINI approach for identifying phenotype-consistent interactions

GEMINI takes in a draft regulatory network and integrates it with the corresponding metabolic network and gene expression data using PROM. PROM uses conditional probabilities, viz. the probability of a given gene being ON or OFF when the regulating transcription factor is ON or OFF, to represent gene states and gene–transcription factor interactions. The ON/OFF state of the TFs is then used to determine the likelihood of an ON/OFF state of the target genes based on the probabilities estimated from microarray data. PROM then utilizes the Gene-Protein-Reaction (GPR) relationships present in the metabolic network models to connect the regulatory targets to the corresponding metabolic reactions. The GPRs take into account the presence of isozymes or multi-gene/multi-subunit complexes that may be involved in catalyzing each metabolic reaction. The probabilities are then used to constrain the fluxes through the metabolic network (detailed below), and an optimal state of the network that satisfies topological and transcriptional constraints is determined.

Using this integrated metabolic-regulatory network, PROM can simulate metabolic phenotypes under different conditions using Flux Balance Analysis (FBA) [28]. FBA identifies the optimal state of the metabolic network that would allow the system to achieve a particular objective, typically the maximization of an organism's growth rate or biomass production. Mathematically, FBA is framed as a linear programming problem:
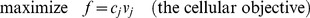
(1)

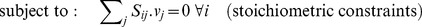
(2)

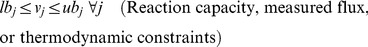
(3)where *i* is the set of metabolites, *j* the set of reactions in the network, *S_ij_* is the stoichiometric matrix, *c_j_* designates the objective function (the cellular growth rate in this case) and *v_j_* is the flux through reaction *j*. PROM finds a flux distribution that satisfies these physico-chemical constraints plus additional constraints to account for the transcriptional regulation [22]:
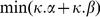
(4)subject to constraints
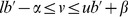
(5)

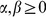
(6)

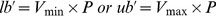
(7)where *lb'* and *ub'* are constraints based on transcriptional regulation and are estimated based on the probabilities. *Vmax* and *Vmin* are the systemic maximum and minimum fluxes through a reaction and are determined using Flux Variability Analysis (FVA) [29]. *α* and *β* represent the deviation from those constraints (determined by the algorithm for each reaction), and κ represents the penalty for such deviations. The higher the value of κ, the greater the transcriptional regulation constraint is on the system. The value of κ is determined in a data-driven manner (See Methods).

Once the initial PROM model is built, GEMINI then performs *in silico* knockouts of each TF in the integrated model and compares the predictions with experimental observations. GEMINI identifies and removes interactions that do not lead to the measured growth phenotype, while retaining the phenotype-consistent interactions. This is achieved by comparing the flux state predicted by PROM for the TF knockout (*v1*) with the closest flux state that represents the measured growth phenotype (*v2*). The flux state *v2* is obtained by forcing the model to match the observed phenotype, while still attempting to satisfy as many of the transcriptional constraints as possible. Mathematically, we solve the same constraints as above with the additional constraint that the predicted growth phenotype matches the observed phenotype (See Methods).

**Figure 1:**
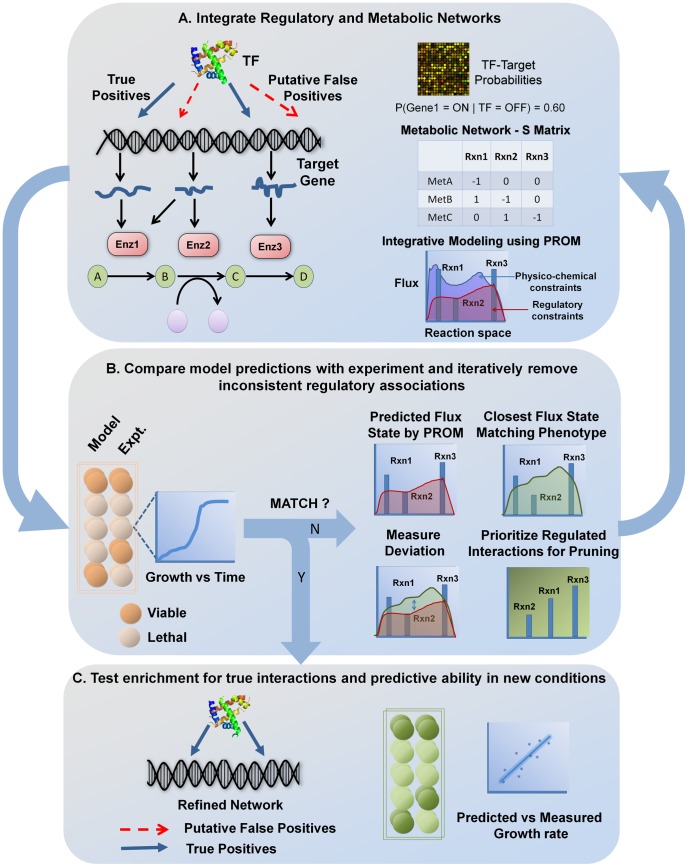
Process of identifying phenotype-consistent interactions using GEMINI. **A.** High-throughput interaction data were mapped onto a biochemically detailed metabolic network using PROM and phenotypic consequences of these interactions were predicted. The metabolic network is represented *in silico* in the form of a stoichiometric matrix, where every column corresponds to a reaction and every row corresponds to a metabolite. The regulatory interactions are represented as probabilities, which are estimated from microarray data. By using constraint-based analysis, it is possible to determine the possible configurations in the biochemical network that correspond to physiologically meaningful states; this is done by applying various physico-chemical constraints, such as reaction stoichiometry and thermodynamics. The interaction probabilities were then used to further constrain the fluxes through the metabolic network and an optimal network state that satisfied both thermodynamic and transcriptional constraints (shaded in red) was determined using PROM. **B.** Interactions that lead to inconsistencies between model predictions and experiments were identified and removed. This was achieved by comparing the flux state predicted by PROM for the TF knockout with the closest flux state that represented the measured growth phenotype; reactions regulated by the perturbed TF were then prioritized based on the magnitude of their deviation. Interactions regulating these reactions were then removed and PROM was run iteratively on each new network to predict the growth phenotype. **C.** The final network that matched the phenotype was evaluated based on its ability to retain known interactions, and predict growth phenotype outcomes in new conditions.

Unlike mass balance or thermodynamic constraints that cannot be violated, PROM imposes “soft” constraints on the system due to transcriptional regulation, thereby enabling us to force the model to match the measured phenotype. This procedure results in a flux solution that is geometrically closest to the flux state *v1*, based on absolute distance, while still satisfying the observed growth phenotype. We then compare the new flux state *v2* with the original flux state *v1*, and prioritized reactions regulated by the perturbed TF based on their magnitude of change. Interactions regulating these reactions were removed consecutively and PROM is run on each new network to predict the growth phenotype. This process is repeated until the inconsistency is resolved (Figure 1).

### Reconstructing an integrated metabolic-regulatory network model for yeast

We demonstrate the GEMINI approach using the model organism *Saccharomyces cerevisiae*. Because of the availability of a large amount of data about regulatory interactions, a vast amount of gene expression and phenotype data, and the existence of a well-curated genome-scale metabolic model for yeast, this organism makes an ideal test case for GEMINI. Most importantly, highly accurate inference of regulatory interactions has been a major challenge in yeast as it is a more complex system than bacterial model organisms such as *Escherichia coli* [1], [30]. To apply our approach to yeast, we downloaded transcriptional regulatory interactions from the Yeastract database [31], which were compiled from various literature sources. These Yeastract interactions have a high-confidence subset (direct/gold-standard interactions) for which strong experimental evidence (supporting the interaction of the TF with the promoter of the specified target gene) is available [31]. This gold-standard subset is commonly used as a benchmark for validating inference algorithms [1]. This dataset allowed us to test our hypothesis that metabolic phenotype-consistency can be used as a criterion for improving the identification of functional regulatory interactions.

The effectiveness of GEMINI was evaluated by measuring its ability to differentiate between the validated direct interactions and the remaining low-confidence interactions (putative/potential interactions), which were inferred using motif search algorithms [32]. It should be noted that the gold-standard interactions are not necessarily perfect and may contain false-positive interactions [1]; similarly, the low-confidence interactions could be either false-positives or true interactions that have not been validated yet. However, on average, the gold-standard interactions have stronger supporting evidence from ChIP-binding or directed mutagenesis—giving them a higher probability of being true than the lower confidence set. According to our hypothesis, gold-standard interactions are more likely to be consistent with phenotype data than the potential interactions. With an unlabeled list of Yeastract interactions as input to GEMINI, what we aimed to test in the refined output network was enrichment for the gold-standard interactions over the potential interactions.

The initial TRN, formed by compiling the Yeastract interactions, was integrated with the yeast metabolic network [33] (composed of 1597 reactions and 901 genes) and gene expression data [34] (consisting of 904 expression arrays in 435 conditions) using PROM (See Methods). 14% of all the interactions in the Yeastract database involved interactions with metabolic genes and the integrated model contains 31,075 interactions between 179 TFs and 863 metabolic genes.

### GEMINI preferentially recalls gold-standard interactions

**Figure 2:**
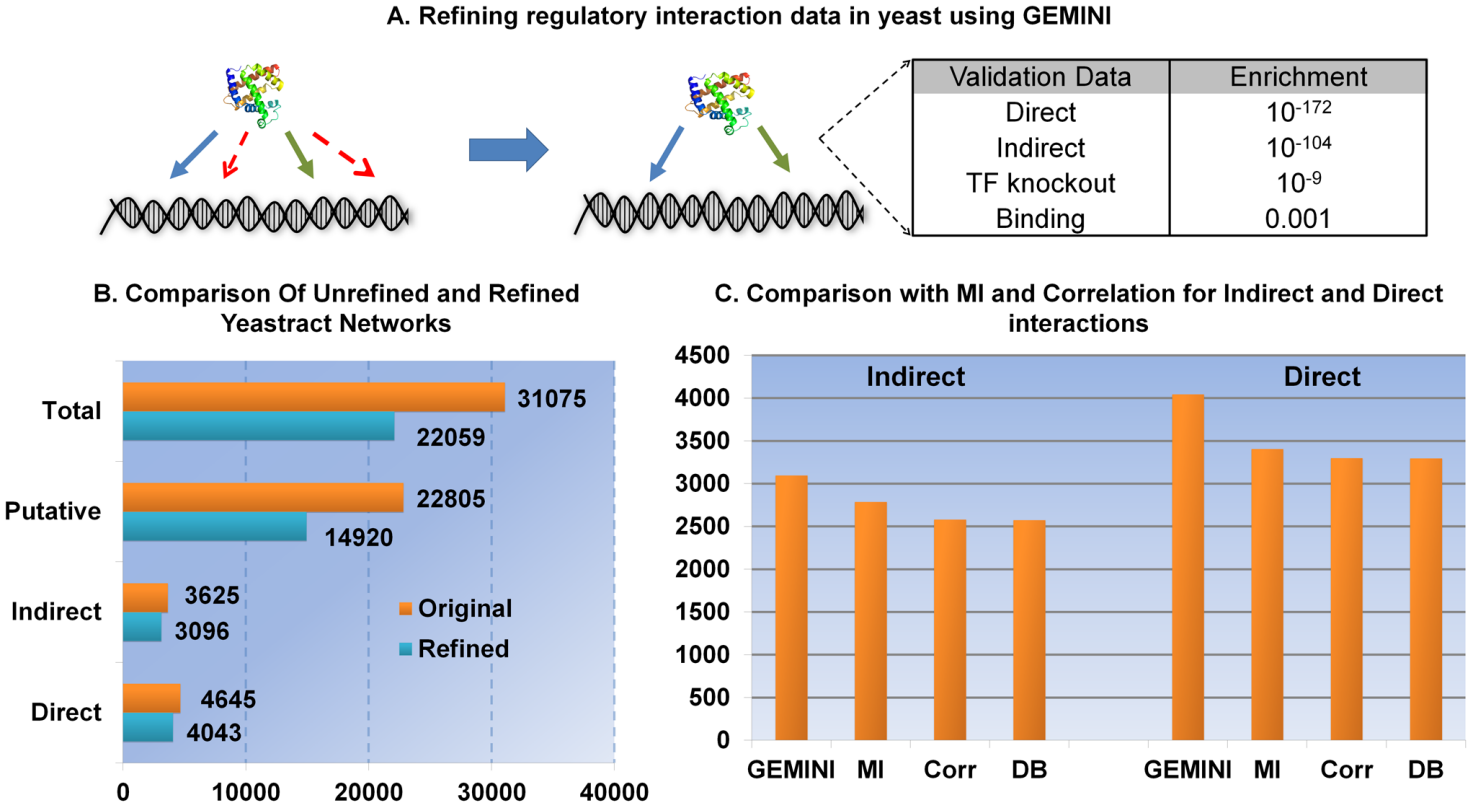
Refining regulatory interaction data in yeast using GEMINI. **A.** GEMINI was evaluated for its ability to preferentially retain the gold-standard interactions (blue edges) and the indirect interactions (green edges). The hyper-geometric p-values for enrichment with various data sets are shown. **B.** Running GEMINI on the network derived using Yeastract resulted in the elimination of ∼9,000 phenotype-inconsistent interactions and produced a refined integrated network model that was more highly enriched for known interactions than the original network (p-value<10^−172^, hyper-geometric test). Most of the interactions eliminated by GEMINI were found to have little supporting experimental evidence (interactions that did have strong supporting evidence were preferentially retained). **C.** The number of true interactions (direct and indirect) recalled was significantly higher than could be recalled using mutual information (MI) or correlation (Corr)-based approaches, which rely on gene expression alone (estimated from the same gene expression dataset and for networks of the same size). We also measured the best prediction obtained by MI and correlation over all possible cut offs and this was still significantly lower than the enrichment obtained by GEMINI. The supplementary figures S1 and S2 show the enrichment for direct interactions over the entire range of thresholds for both MI and correlation. The number of interactions recalled by random sampling from the Yeastract database (DB) is also shown, as a reference.

GEMINI performed *in silico* knockouts of each TF in the model and compared the predictions (i.e., lethal or viable) to data from growth viability assays in glucose minimal media [35]. Running GEMINI on this network eliminated over 9,000 phenotype-inconsistent interactions and results in a final network containing 22,059 phenotype-consistent regulatory interactions. In comparison to the original YEASTRACT network, we found the final integrated network built using GEMINI to be highly enriched (p-value = 10^−172^, hyper-geometric test) for validated gold-standard interactions; this result suggests that GEMINI preferentially removed low-confidence interactions (Figure 2).

These results were robust to the chosen growth conditions – glucose, galactose, glycerol and ethanol minimal media all led to significant enrichment of gold-standard interactions (Table 1). We also observed the same effect when we did the same analysis using a different metabolic network model (iMM904; See Methods), regulatory networks from different sources (binding, motif-based and expression-based inference; see section below), different subsets of the Yeastract TRN (Figure S4) and using different metrics to prioritize interactions (Figure S9).

**Table 1:** Enrichment across different carbon sources.

Condition	Enrichment for Direct	Enrichment for Indirect	Final Network Size
Glucose	10^−172^	10^−104^	22059
Galactose	10^−96^	10^−55^	22308
Glycerol	10^−179^	10^−100^	22134
Ethanol	10^−144^	10^−86^	22551
Rich/undefined Media	10^−42^	10^−39^	28981

Using growth viability information from different environmental conditions (rich media and minimal media with galactose, glycerol, and ethanol as the carbon source, respectively) had a similar effect on the network refinement. Generally defined minimal media were more useful than rich media, which provided the least enrichment for gold standard interactions. Importantly, there was considerable overlap in the interactions retained by running GEMINI in each condition.

### Comparison with gene expression-based metrics

To determine whether a similar accuracy could have been obtained using expression data alone (i.e., without adding constraints based on the phenotypic outcomes predicted by the metabolic network), we compared our GEMINI results to a more commonly used approach for curating TRNs—sorting predicted interactions based on the correlated expression of the TFs and their putative target genes. Specifically, we measured the Mutual Information (MI) and Pearson's correlation among all of the interactions in our original YEASTRACT network.

To ensure comparison was not biased towards GEMINI, we tuned the size of the network using MI and correlation over all possible values (over-fitting to the best outcome that could be achieved for MI or correlation for any cutoff). The maximum enrichment obtained by MI and correlation (even when overfit) was lower than that obtained using GEMINI (the lowest p-value measured over all possible network sizes for MI was 10^−6^ and for correlation was 10^−3^; Figure S1 shows the enrichment obtained over the entire range of thresholds for both MI and correlation). The high enrichment obtained by GEMINI strongly supports our hypothesis that additional phenotype data and integration with the biochemical details represented through the metabolic network can be used as an effective constraint to refine high-throughput interaction data.

To gain further insight into the types of interactions recalled by the different methods, we examined another subset of interactions having “indirect evidence”—interactions inferred based on changes in the mRNA or protein expression of a target gene after perturbing its putative regulator [31] (Figure 2). MI and correlation performed significantly better at recalling indirect interactions than direct interactions (p-value of 10^−19^ and 10^−4^ for the best cutoffs of MI and correlation, respectively); this is not surprising since the indirect relationships are defined by gene expression changes. However, GEMINI still outperformed these methods in recalling indirect interactions (p-value of 10^−104^) for any network size (Figure 2c and Figure S2). Therefore, GEMINI seems to more effectively distinguish both evidence-based direct and indirect interactions from a background of lower-confidence inferred interactions. Furthermore, no significant difference in the distributions of the MI scores was observed between the interactions retained and removed by GEMINI based on the Kolmogorov-Smirnoff test, showing that the phenotype data and integration with the metabolic network provides significant independent information (Figure S3).

### Refined model is consistent with gene knockout phenotypes, gene expression data and the metabolic network

The biological relevance of the interactions retained by GEMINI is also supported by the enrichment for biological processes relevant to the set of target genes for each regulator. As compared to regulons (target genes for each regulator) in the original network, regulons in the refined network were found to be more specific, on average, to a given metabolic pathway (p-value<0.01; Methods). The number of enrichments for specific metabolic pathways increased from 165 to 184 despite the removal of over 9000 interactions, suggesting that the phenotype-consistent regulons identified by GEMINI are associated with a more coherent set of molecular and metabolic functions, and most TFs tend to regulate distinct cellular processes as has been observed previously [3], [8], [36]. Through this process of refinement, we identified new statistical associations between TF and specific metabolic pathways (Table S1). More interestingly, GEMINI removed an association between the TFs, Msn4 and Gis1, and the TCA cycle. The availability of flux measurements for the knockout strains of these two TFs enabled us to validate this prediction. Comparison with C13 flux data [13] showed that the knockout of these TFs did not in fact affect the flux through the TCA cycle.

Comparison with TF knockout expression data from a recent study [15] also supported the functional significance of the phenotype-consistent interactions. This expression set was not part of the microarray compendium used for running GEMINI and allowed us to assess the predictive ability of the phenotype-consistent interactions. For 152 transcription factors in our network, we obtained a list of genes that were differentially expressed after the TF was knocked out (FDR<0.05; [37]). We compared this list with the list of predicted target genes in the original Yeastract network and the refined network. We found that the targets of TFs in the refined network were more likely to be differentially expressed than those in the original network when their corresponding TF was knocked out (p-value = 10^−9^; Methods). While we had selected interactions based on their consistency with phenotype, their ability to match expression changes in new conditions provided additional support for GEMINI. The phenotype-consistent interactions also had higher TF-DNA binding affinity than the original network (p-value = 0.01; t-test; Methods), as measured from protein binding microarray (PBM) data [38]. These results also provide additional evidence supporting the validity of the potential interactions that were predicted to be phenotype-consistent by GEMINI. This suggests that GEMINI is effective at identifying functional interactions and is consistent with various heterogeneous data.

### Iterative approach for network refinement and phenotype prediction

One interesting observation from our results is that GEMINI can differentiate interactions from different sources based on their effect on the predicted phenotype. We next checked to see if we can use this to evaluate newly inferred interactions in the context of available known interactions. We can subsequently reconcile inconsistencies that arise from these interactions with metabolic phenotypes. To simulate such a scenario, we added new interactions onto the refined Yeastract network model and refined the expanded network model using GEMINI.

We chose three commonly used data types:

- Interactions inferred based on sequence motif search learned from ChIP [39] (Network I);
- Interactions inferred using the expression-based reverse engineering algorithm, CLR [4] (Network II);
- Validated direct and indirect interactions in the literature measured using experiments such as large-scale TF knockout [15], [37], PBMs, and ChIP-chip [38], [40] (Network III).

We found that for both the motif and CLR network, we could refine the network further and significantly enrich once again for direct and indirect interactions (enrichment p-value compared to the original inferred network (direct, indirect) = (10^−44^,10^−73^) and (10^−13^,10^−31^) for motif and CLR, respectively; Table 2). A wide variety of reverse engineering algorithms have been developed recently to infer potential regulatory interactions from sequence, gene expression data [1], [4], [8] or through integration of various data types [2], [8]. These algorithms rely on correlated patterns of expression or the occurrence of a sequence motif in the upstream region of the target gene [5]. The enrichment for gold-standard interactions suggests that GEMINI could be integrated with these network inference and reverse engineering approaches to improve the identification of functional regulatory interactions. This result is consistent with the observation that an integrative network inference approach combining heterogeneous omics data could lead to more predictive TRN models [2]. While inference approaches like CLR allow for predicting potentially new TF-gene interactions, GEMINI is a refinement algorithm and it is not an alternative to these de novo inference approaches, but may be used in conjunction with such approaches to enhance their prediction by combining orthogonal data types. Overall, this result provides additional validation that GEMINI works across multiple data sets from different sources.

**Table 2:** Network sizes and the number of interactions retained after running GEMINI for each network type.

Data Set	Enrichment for Direct	Enrichment for Indirect	Network Size (Initial/Final)
I. Motif data	10^−44^	10^−73^	38105/28807
II. Expression (CLR)	10^−13^	10^−31^	24111/21954
III. Validated interactions	NA		29874/29808
Validated interactions (Quantitative Iteration)	10^−27^		29874/25000

The hyper-geometric enrichment p-value compared to the original inferred network is shown. Note that for Network III with validated interactions, a single p-value was obtained because we were unable to differentiate between direct and indirect interactions in some of the new interactions that were added. So a single p-value for validated interactions was obtained.

**Figure 3:**
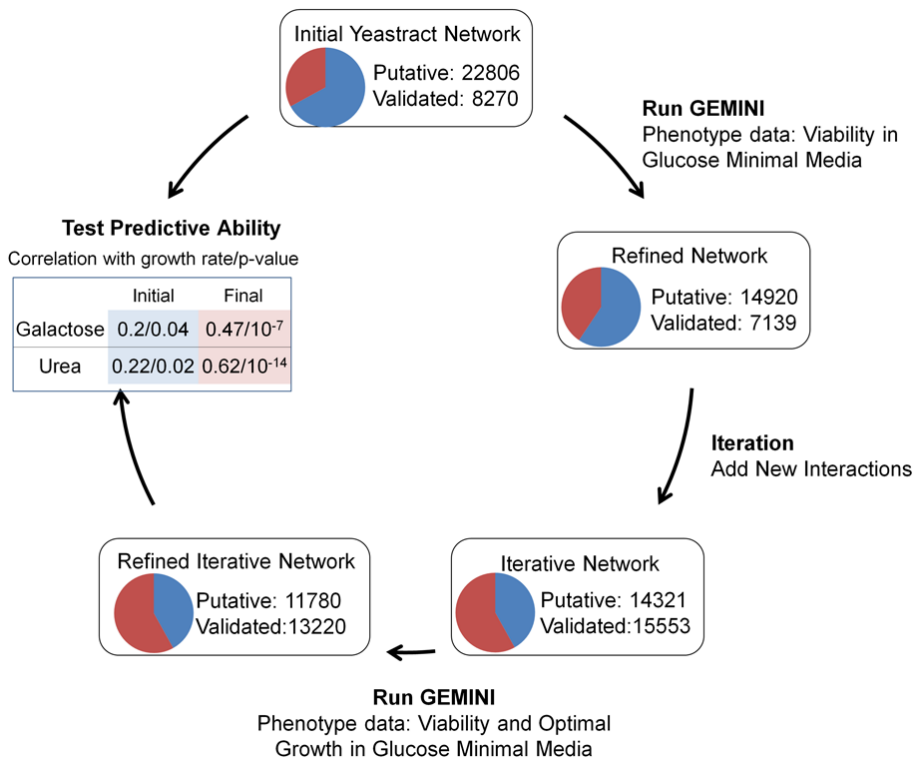
Iterative approach for network refinement and phenotype prediction. By using an iterative approach, we increased the comprehensiveness of the integrated network model by adding new interactions (Network III) and iteratively refining the model using GEMINI. This process enriched the fraction of validated interactions in the network (shown in red) and improved the predictive ability of the integrated network model.

In contrast to the inferred interactions, very few interactions (∼66) from the validated interaction data set (Network III) were removed by GEMINI. This interaction set is similar to the gold-standard set in the Yeastract database and was thus retained in the network. While these interactions were consistent with the simple lethal/non-lethal constraint we used in glucose minimal media, we predicted that by adding more constraints, we could narrow down the solution space further, and remove more phenotype-inconsistent interactions. With this aim, we employed PROM to quantitatively predict the growth rate (as opposed to just lethal/non-lethal outcomes). Doing so allowed us to partition the non-lethal predictions into two categories: suboptimal and optimal (Methods). Using this strategy for the 118 TFs in our network for which experimental measurements of this kind were available for comparison [13], we were able to eliminate 4874 more interactions, while still improving the enrichment for the validated interaction set (p-value of 10^−27^; Table 2; Figure 3).

Importantly, we observed that the refined network had a greater consistency with growth phenotype data in new conditions than the original network. Thus, by learning only on glucose minimal medium, the network model had greater correlation with growth rate measurements in galactose minimal medium (correlation of 0.47, p-value = 10^−7^ vs. a correlation of 0.2, p-value = 0.04 for the original unrefined Yeastract model) and in urea minimal medium (correlation of 0.62, p-value = 10^−14^ vs. a correlation of 0.22, p-value = 0.02; data from Fendt *et al.* [13]). This is not unexpected because we were removing inconsistencies in one condition, which may have produced the same discrepancy in the other conditions as well. Nevertheless, the result suggests that GEMINI improves the overall predictive ability of the integrated regulatory-metabolic network model under new environmental conditions (Figure 3). We were also able to expand our integrated network model from 22,059 to 25,000 interactions through the addition of this validated interaction set.

## Discussion

In this study, we developed a novel way to connect regulatory interactions with phenotype data using a metabolic network. Currently, accurate regulatory network reconstruction is hampered by the lack of methods to directly connect inferred potential interactions to observable phenotypes such as growth rate to guide the inference of these networks in a high-throughput fashion. Using GEMINI, we demonstrated that we can identify functional regulatory interactions and refine high-throughput interaction data using phenotype-consistency as a constraint. We showed that by integrating with a predictive metabolic network model, we can improve the quality and predictive ability of the generated high-throughput data significantly better than using gene expression alone.

### Resolving phenotype inconsistencies

By applying the GEMINI approach to our yeast model, we identified phenotype inconsistencies for 80 TF knockout predictions. The majority of the inconsistencies (85%) were of the type NGG (No Growth – Growth), for which the model predicts lethality (or suboptimality), while the actual phenotype was non-lethal (or optimal). Because this scenario was the most commonly identified inconsistency type, we concentrated on reconciling this set alone. Also, this case is more tractable to resolve than the opposite case (GNG), which involves adding interactions from a very large multi-optimal solution space. Further, a TF knockout may be lethal or suboptimal due to a non-metabolic reason, meaning that even an optimal metabolic model would not be expected to resolve all GNG inconsistencies; in contrast, if a knockout is non-lethal and the model predicts it to be lethal, then that implies there is an inconsistency with the integrated model.

GEMINI integrates two different network models (metabolic and regulatory) and inconsistencies could arise due to either network. In this work, we assumed that the metabolic network, being better curated and having a biochemical basis, could be used to identify inconsistencies in the regulatory network. Additional evidence from the distribution of inconsistencies also supports our assumption (Figures S4 and Figure S10; discussed below). Furthermore, NGG inconsistencies arising due to the metabolic model were circumvented by using a metabolic model in which the GrowMatch algorithm [21] was run to resolve the NGG inconsistencies (Zommorodi and Maranas model [33]). To test the sensitivity of our approach to the metabolic model used, we repeated our analysis with an older version of the metabolic model (iMM904 [41]), which has a lower predictive accuracy than the Zommorodi and Maranas model. We found that even with iMM904, GEMINI was able to strongly enrich for direct interactions (p-value = 10^−104^), but not as strongly as when using the more predictive model by Zommorodi and Maranas. This suggests that as the predictive ability of the metabolic models improves, we should be able to refine these interactions further. In theory, a trivial solution for resolving NGG inconsistencies is to remove all of the interactions for the respective TF. However, interestingly, GEMINI resolved all 80 NGG inconsistencies without reverting to the trivial solution.

Furthermore, the elimination of phenotype inconsistent interactions by GEMINI based on one condition might lead to inconsistent predictions in a different condition. We found that this was the case for a small fraction (4%) of the interactions that were phenotype-inconsistent in glucose minimal media, but were predicted to be consistent with growth phenotype data in galactose minimal media. Analyzing inconsistencies over different set of conditions would help us avoid over fitting the model to the growth phenotype data. Further analysis across conditions would help uncover interactions that are condition-specific and post-transcriptionally regulated (discussed below).

### The role of network size and topology

In the present analysis, we used the predicted growth rate as the only phenotype to constrain the regulatory network. If the interactions regulating biomass-related metabolic reactions were enriched for potential interactions, this would lead to an apparent enrichment for direct gold standard interactions on running GEMINI as an artifact. We tested this by evaluating the metabolic genes for which their knockout affected the maximum growth rate of the model. No difference was observed in the number of gold-standard interactions regulating this set of genes versus the rest (both the sets had the same fraction (14%) of gold-standard interactions; Methods). A similar distribution of gold-standard interactions was also found for interactions regulating dead-end reactions that do not contribute to the biomass and the rest of the metabolic network. Hence, there were no apparent underlying biases in the metabolic network architecture that led to the enrichment of gold-standard interactions.

We predicted that the effectiveness of GEMINI would also depend on the scale of the regulatory network model used. GEMINI evaluates interactions in the context of other interactions in the network and so its effectiveness will depend on the size and degree of completeness of the entire network. To test this, we ran GEMINI using different fractions of the entire TRN and looked at the enrichment for gold-standard interactions. As expected, we found that GEMINI's effectiveness to refine the network increased with the size of the input network. To control for size bias on the enrichment p-value, we also looked at the fraction of gold-standard interactions in the initial and final refined network and observed the same effect (Figure S4).

### Inconsistencies highlight incomplete biochemical knowledge

**Figure 4:**
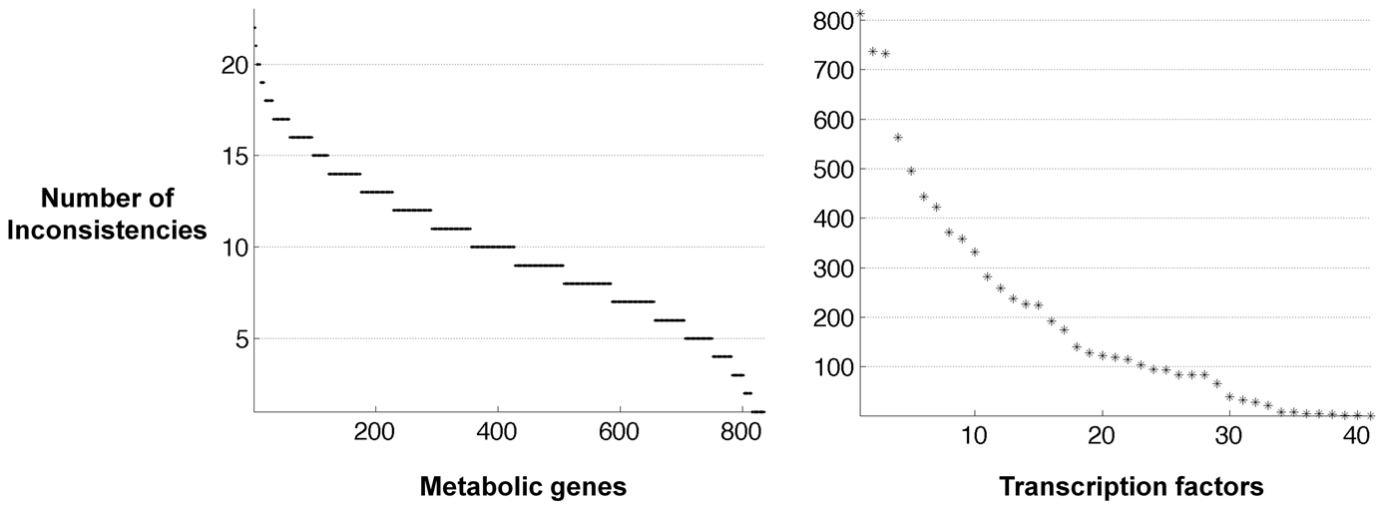
Distribution of inconsistencies across the regulatory and metabolic network. The distribution of phenotype inconsistencies was exponential across the TRN, suggesting that a few TFs led to most of the inconsistencies. In contrast, the distribution of inconsistencies across the metabolic network was linear and did not reveal any strong trend towards specific metabolic genes.

GEMINI utilizes the mechanistic information in biochemical networks to refine high-throughput interaction data. We next sought to determine which parts of the yeast transcriptional regulatory network were prone to inconsistencies across different growth conditions (Table 1). We analyzed the distribution of inconsistencies among the 41 TFs that led to inconsistencies using the qualitative phenotype data across the four carbon sources. The distribution was approximately exponential suggesting that a few TFs led to most of the inconsistencies (Figure 4). By identifying key regions that lead to the most inconsistencies, we can prioritize experiments to refine the regulatory network. Further, it highlights regions that are prone to inconsistent predictions while analyzing integrated network models. The top three TFs with most inconsistencies were Ash1, Fkh1 and Fkh2; Ash1 encodes a transcription factor that is involved in mating type switching and while the genes Fkh1/2 are involved in cell cycle regulation. Interestingly, all these TFs have important roles outside metabolism suggesting that the interactions with metabolic enzymes might be false positives due to sequence-based inference.

In contrast to the regulatory network, analysis of the distribution of inconsistencies across the metabolic network did not reveal any strong trend towards specific metabolic pathways. The distribution was linear rather than exponential across the metabolic genes (Figure 4). This suggests that relative to the regulatory network there were no specific genes in the metabolic network that were much more prone to inconsistencies. This is consistent with our previous observation that no underlying biases in the metabolic network architecture led to the enrichment of gold-standard interactions. Among the metabolic pathways highlighted in Table S1, the pentose phosphate (PP) pathway had the most number of inconsistencies. Being a well-studied pathway in yeast and other organisms, it's more likely that the inconsistency arose due to the regulatory interactions rather than due to the PP pathway.

Among the carbon sources, galactose led to the least enrichment for both validated gold standard interactions and indirect interactions. Both glucose and galactose enter central metabolism at the level of glucose-6-P, but they lead to primarily fermentative or respiro-fermentative metabolism, respectively [13], [42] This suggests that we have perhaps incomplete knowledge about the regulatory network changes that happen during growth in galactose, though extensively studied [13], [43], [44], and despite being similar at the metabolic level to glucose. GEMINI also performed poorly on rich media, which is primarily due to the limitations in the representation of the media constituents in rich media within a constraint-based modeling framework.

### Inferring post-transcriptional regulatory mechanisms

Analysis of phenotype-consistent interactions inferred using GEMINI under different environmental conditions (Table 1) revealed potential post-transcriptional regulatory mechanisms. Although there was considerable overlap between the phenotype-consistent interactions predicted from different minimal media conditions, we identified 1170 interactions that were phenotype-inconsistent in only one condition, but were retained in all the other three conditions (Table S2). The fraction of direct and indirect interactions among the 1170 interactions was quite similar to those interactions that were retained in all conditions. We predicted that these interactions might be true interactions that are conditionally-inactive, and the phenotype inconsistency might have arose due to post transcriptional regulatory mechanisms inactivating these interactions in these conditions. While the static information from the gene regulatory network and gene expression predicted the interactions to be active, combining this information with phenotypic data resulted in identifying post-transcriptional regulatory mechanisms that may have turned off these interactions.

Glucose repression is one of the most well-studied processes in yeast and we focused on a subset (408) of these 1170 interactions that were predicted to be inactive only in glucose minimal media. The top 3 TFs with most interactions in this list—Rph1, Hsf1 and Adr1, were all activated during glucose starvation and are regulated via signaling and phosphorylation [45] [46] [47]. For example, the TF Hsf1 is constitutively phosphorylated, but under glucose starvation, it becomes hyper-phosphorylated and adopts an activated conformation resulting in the transcription of target genes [45]. The other TFs are activated through similar mechanisms in the absence of glucose. This is consistent with our prediction that the interactions that lead to inconsistencies only in glucose media were true interactions that are conditionally-inactive in the presence of glucose. Thus, we can potentially infer interactions that are not transcriptionally mediated through this approach. The condition-specific predictions also agreed well with a list of manually curated TF-environment interactions from the regulatory network model of Herrgard et al. [19] for 6 of the 7 predicted glucose-repressed TFs that were present in both the models.

This strategy shows the utility of looking across multiple conditions to identify discrepancies in the data, which might be due to additional biological regulation. This also highlights the importance of incorporating signaling networks as they become available into these integrated network models.

### Expansion and applicability of GEMINI to other systems

Given the large amount of data required to run GEMINI, we are currently restricted to a few well-studied systems with adequate expression, knockout phenotype and network data. However, with the development of automated methods for reconstructing metabolic networks [48], GEMINI could be used as part of a network inference pipeline to identify functional regulatory interactions that are inferred from omics data, and reconcile the interactions with metabolic phenotypes for a large number of sequenced organisms. Another limiting factor in this study was the phenotype data used for analysis. The use of gene deletion growth phenotype data in the current study might restrict GEMINI's application only for microbes for which such a knock-out library exists and has been measured in great enough detail across different conditions. This approach might not be feasible for use in higher organisms like humans and mice. Yet, in theory, phenotype data other than that from growth assays such as metabolite uptake or secretion could be used to limit the space of possible functional states of the TRN and could be applied to higher organisms.

The regulatory network model used in this study, despite being genome-scale and much more comprehensive than the current integrated model for yeast [19], does not comprise the entire TRN. We have focused only on a subset (14%) of the TRN that regulate metabolism. Nevertheless, this subset of the TRN is very well studied and has important applications in metabolic engineering and synthetic biology. Further, the scale of the TRN is primarily limited by the size of the biochemical model with which it interfaces. Although we have restricted our analysis to interactions involving metabolic reactions in the present work, the GEMINI approach is generally applicable to other cellular network types [25], [49], such as signaling networks, as they become available. By integrating other network types, one might account for additional regulatory-phenotype relationships and thus improve predictions even further.

### Conclusion

Regulatory network inference is a significant challenge today as the system is underdetermined and often results in multiple models that could explain the same data with equal efficacy. Thus, it is important to incorporate diverse heterogeneous data types like expression, binding and growth phenotype to constrain the solution space. GEMINI exploits this principle to refine high-throughput regulatory interaction data and identifies interactions that are consistent with various data types. Importantly, this is the first such approach that ties the inference of a transcriptional regulatory network from high-throughput data with a biochemically detailed metabolic network.

We believe this to be an important first step towards mechanistically refining a network model of one type (gene regulatory) using data from another network type (metabolic). Further, our approach highlights the potential of using a biochemically-detailed mechanistic framework to interpret high-throughput data and identify and reconcile inconsistencies across different data types. We find that the data types that are more consistent with each other also have greater evidence supporting their existence. While there are still several challenges ahead for regulatory network inference, the methods presented here lay the foundation for the rapid refinement of omics data using a mechanistic framework, which will advance the study of metabolic regulation and lead to better predictive models of the cell.

## Methods

### Comparison with experimental phenotype data

Using PROM, we predicted the growth outcome of knocking out each TF in the network under a specific condition. By comparing our simulations with experimental growth viability data, we identified and reconciled inconsistent predictions. TF knockouts were predicted to be lethal if the respective maximal growth rate prediction of the mutated organism was less than 5% of the wild-type growth rate [22], [50]. Any knockout that resulted in a growth-rate lower than 95% of the wild-type was considered suboptimal, as has been used previously in other analyses [22], [26]. These results were robust to the choice of the growth thresholds (Figure S5). While we used the values commonly used in the literature, tuning these thresholds indicated that higher enrichments could be achieved by varying this parameter. However, we recommend using the default values to avoid over-fitting.

### Inferring the closest flux state to the measured phenotype

The closest flux state that represents the measured growth phenotype (*v2*) was obtained by solving the same optimization problem for PROM with the additional constraint that the predicted model growth rate matches the observed growth phenotype:
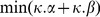
(8)subject to constraints
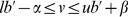
(9)

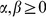
(10)


*Additional constraint –*

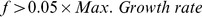
(11)
*or*

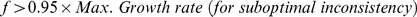
(12)
*f* is the predicted growth rate by the model, and 0.05 and 0.95 are the growth rate thresholds for determining viability and suboptimality as mentioned above. The solution obtained by solving this above problem gives flux state *v2*.

The entire steps in GEMINI are described in the pseudo code below:

- *Build PROM model*

*For each TF {*

1. *Run PROM for the TF knockout*
2. *Find inconsistent predictions i.e predicted growth rate/phenotype≠measured growth rate/phenotype*
3. *IF NGG (No Growth (Predicted Phenotype) – Growth (Measured Phenotype))*
  - *Note the flux vector (v1)*
  - *Force the model to match the measured phenotype (section above)*
  - *Note the new flux vector (v2)*
  - *Sort reactions (R) regulated by the TF based on magnitude of flux change between v1 & v2*
  - *Find the reaction (R) regulated by the TF whose fluxes change the most between v1 & v2*
  - *For this reaction (R) find the corresponding gene(s)*
  - *The interaction between the TF and the corresponding gene(s) is removed*
  - *Get a new regulatory network*
4. *Run PROM for the TF knockout and IF phenotype matches experiment, Output the final regulatory network*

*ELSE REPEAT from 3e till it matches actual phenotype*

*}*

### Alternate optima for flux state v2

The flux solutions in FBA have multiple possible states, while the growth rate or the objective function is unique. Since we relied only on the growth rate and the transcriptionally constrained reactions (part of the objective function in PROM) as the metric to refine the network, the final network structure was identical across different runs of GEMINI. To further investigate how alternate optimal solutions alter the effectiveness of GEMINI we generated new flux solutions by introducing small changes to the growth threshold (step 3b in pseudo code and equation 11). We compared five different networks across different combinations generated by changing the growth threshold. This generated new flux solutions with approximately the same growth rates. We found that the networks were 99.9% similar across these small perturbations that led to alternate flux solutions (Table S3). We can infer that the same subset of phenotype-inconsistent interactions is removed across various growth thresholds and flux optima. The use of regulatory-constrained reactions in the objective function in PROM ensures that there is no variability between runs and we get the same solution each time while running GEMINI. Furthermore as mentioned earlier, strong enrichment for validated interactions were obtained over a wide range of these growth thresholds (Figure S5).

### Alternate optimal solutions for the refined regulatory network

The above analysis of inferring regulatory networks across alternate metabolic flux solutions also resolves the possibility of multiple alternate optima with respect to the regulatory network. We found that the same core set of interactions was removed across different runs. In addition, we also compared network generated using much larger changes in growth rate threshold used for inferring the flux state v2. We once again found that while the refined network sizes changed across different thresholds, they were >95% similar to each other among the interactions that were retained. These results indicate that that there is a strong global optimal state for the regulatory network and by perturbing the model and constraints we still converge close to the global optima. In terms of network refinement, all these results suggest that there is a core set of regulatory interactions that are removed across different constraints and conditions (Table S3). While its still certainly possible that there are multiple other optimal flux and regulatory network solutions, the use of regulatory-constrained reactions in the objective function in PROM ensures that there is no variability between runs and we get the same solution each time while running GEMINI.

### Estimating the penalty factor κ

The value of κ, which determines the strength of the transcriptional regulatory constraint, was determined in a data-driven manner by tuning across a range of values. We set κ to be the lowest value above which there was no increase in the number of interactions removed (Figure S6). We obtained a κ value of 10 for all of our simulations based on this strategy. The results were robust to the value of κ chosen this way for a wide range of values above 10 (Figure S7).

### Alternative approaches to prioritize interactions

We have used a metabolic network-based approach for prioritizing regulatory interactions for pruning. One can envision other approaches and metrics to prioritize these interactions. As an alternative metric, we sorted interactions based on probabilities instead of predicted flux difference (see step 3 in the pseudo-code). While this seems to be a straightforward metric, this ignores the system-level effect of these interactions on the biochemical network for prioritizing the interactions. Using this approach on the yeastract data, we obtained an enrichment of 10^−20^ for direct interactions. Note that even though we only use transcriptomic data to prioritize interactions, this approach yields higher enrichment than MI or correlation. This is because we prune interactions till the predicted systems-level growth phenotype matches the experimental measurement; thus the systems level constraint is unchanged while only transcriptomic data is used for prioritizing interactions.

As a second alternative approach for prioritizing interactions, instead of sorting interactions based on the flux difference between the predicted (v1) and expected (v2) flux state, we assigned the reactions into two groups – the first group of reactions change significantly based on a z-score threshold between v1 and v2 and the rest that did not change significantly. Interactions that regulate these reactions were then pruned randomly from the first group and then from the second group. The rationale being that this strategy doesn't rely significantly on the absolute difference between reactions and allows for alternate flux solutions. We once again found strong enrichment for gold standard interactions through this approach across different thresholds (p-value = 10^−143^). This method is further discussed in Figure S9. The strong enrichments using different metrics and thresholds suggest that the systems level constraints are relatively more important than the order in which the different inconsistencies are solved.

### Robustness to various inputs

Both expression randomization and phenotype swapping removed the enrichment for gold-standard interactions (p-value = 1). We also performed bootstrapping of expression data to determine sensitivity to the gene expression data used. This was done by running GEMINI using random subsets comprising 80% of the expression data. We found strong enrichment in all of the runs (p-value<1E-90; Figure S8), suggesting that the data were sufficiently powered for this analysis.

All parameters were left at the default value as recommended for running PROM (binarization threshold – 0.33 i.e. the 33^rd^ percentile of gene expression data (Figure S10); lethal/non-lethal growth threshold – 0.05 (Figure S5)). The parameter Kappa is determined in a data driven manner by the GEMINI algorithm as mentioned above. We found that much higher enrichment could be achieved by changing the binarization threshold (upto 10^−220^; Figure S10); nevertheless, we recommend using the default parameter values while running GEMINI to avoid over fitting.

### Biases in the metabolic network architecture

For the analysis to identify potential biases in the network architecture, we identified genes affecting maximal growth rate by doing a systematic single gene deletion of all the metabolic genes in the model in glucose minimal media. We identified interactions that regulate this set of genes and compared it with the rest of the interactions in the network. We found the fraction of gold standard interactions to be the same in both sets of interactions. Dead end reactions used for this analysis were identified using the removeDeadends algorithm in the COBRA toolbox in MATLAB.

### Metabolic network and growth conditions

We used the reconstructed yeast metabolic network by Zommorodi and Maranas because it had the highest predictive ability among the available yeast models [33]. In our simulations, the carbon source and oxygen uptake were constrained to 10 mmol/h/gDW and 2 mmol/h/gDW, respectively. Ammonia, phosphate, and sulfate were assumed to be non-limiting. Trace amounts of essential nutrients that were present in the experimental minimal media formulation (4-aminobenzoate, biotin, inositol, nicotinate, panthothenate, and thiamin) were also supplied in the simulations. Flux variability analysis for PROM was performed using the FastFVA algorithm [51].

### Gene expression data

Robust multi-array averaged (RMA)-normalized gene expression data consisting of 904 arrays in 435 conditions were obtained from the Many-Microbes Microarray Database [34]. This microarray compendium was chosen with the aim of maximizing the number of conditions under which gene expression is measured, while reducing array platform-induced variations [22], [52].

### Regulatory network data

All the regulatory interaction data were obtained from the supplementary material of the respective publications [37] or from the author's website [38], [40] and from the YEASTRACT database [31]. Among these interactions, only those involving metabolic genes, and those that had corresponding expression data in the Many Microbes Database were retained.

### Growth phenotype data

Growth phenotype data for yeast TF knockout strains grown in glucose, galactose, glycerol and ethanol minimal media were obtained from Kuepfer et al [35]. These data provided a list of lethal/non-lethal predictions under different conditions. Quantitative growth data were obtained from Fendt et al [13] in glucose, galactose and urea minimal media. TFs with missing values in the Kuepfer et al or Fendt et al phenotype data were not refined using GEMINI.

### Estimating biological and functional significance

Metabolic pathway enrichment analysis was done by overlapping genes in each regulon with genes in each pathway (like TCA cycle or glutamate metabolism) as defined in the metabolic network model. The p-value for overlap between the regulons and pathway genes was calculated using the hyper-geometric test.

In the analysis to determine the functional significance of the interactions, the differentially expressed genes (FDR<0.05) were obtained from Reimand *et al.* [37] based on their analysis of a comprehensive TF knockout experiment by Hu *et al.* [15].

For the comparison with PBM data [38], we compared the distribution of interaction ranks for the original and refined network. We used a t-test to test the hypothesis that the mean rank for the refined network was lower than the mean rank of interaction for the original network (p-value = 0.001).

### Networks for iterative refinement

The sequence motif data were obtained from the supplement of MacIsaac *et al.* [39]. A TRN model was inferred using the algorithm CLR [4] with default parameters (number of bins = 10 and spline degree = 3), and using the expression data from the Many Microbes Database. Predicted interactions with z-scores greater than two (mutual information greater than two standard deviations above than background) were chosen in the final network. Interactions involving metabolic genes were then identified and used for the analysis. For the TF knockout data described previously, the top 100 genes with the lowest p-value (below a FDR threshold of 0.05) were considered to be targets for each TF. This was done to limit the size of the TRN. Table 2 gives the network sizes and the number of interactions retained in each case.

### Statistical analysis

Mutual Information between interactions was measured using the algorithm ARACNE [7] with default parameters. The p-value for overlaps and enrichments with different interaction sets was calculated using the hyper-geometric test. We calculated a p-value for each comparison by summing over probabilities for all values of overlap> = L, the length of the overlap. The obtained p-values were rounded off to the closest power of 10 for clarity.

All the simulations and statistical analyses were performed in MATLAB. The COBRA toolbox [53] was used to load and optimize the metabolic model. The optimization problem was solved using the GNU Linear Programming Kit (GLPK) solver. The GEMINI algorithm along with a faster version of PROM, and the integrated metabolic-regulatory network models for yeast, are available for download at https://sourceforge.net/projects/gemini-data/.

## Supporting Information

Figure S1 Mutual Information (blue) and correlation (red) tuning across various network sizes. Plots show enrichment (shown as the negative log to the base 10 of the hypergeometric p-value) for direct interactions. The same gene expression data set used for GEMINI (904 arrays in 435 conditions) from the Many-Microbes Microarray Database were used for estimating MI and correlation. Interestingly, we observed that redoing the same analysis using interactions with positive pearson's correlation alone yielded higher enrichments (minimum p-value = 10^−10^), while interactions with negative pearson's correlation did not lead to any enrichment for direct interactions (minimum p-value = 0.99).
(TIF)

Figure S2 MI (blue) and correlation (red) tuning across various network sizes for indirect interactions. Plots show enrichment (shown as the negative log to the base 10 of the hypergeometric p-value) for **indirect** interactions. Interestingly, similar to direct interactions, looking at interactions with positive pearson's correlation alone yielded higher enrichments (minimum p-value = 10^−16^), while interactions with negative pearson's correlation did not lead to any enrichment for indirect interactions (minimum p-value = 0.96).
(TIF)

Figure S3 Comparison of the distributions of the MI scores for the original and refined yeastract networks. We found that the interactions retained by GEMINI do not consist only of the lower part of the total MI distribution, except for extremely low MI values close to zero. The pruning of the network by GEMINI is less trivial than simply raising the threshold to select for significant MI scores. The Kolmogorov-Smirnoff test also revealed no significant difference (p-value = 1) between the two MI distributions.
(TIF)

Figure S4Effect of the size of the input TRN 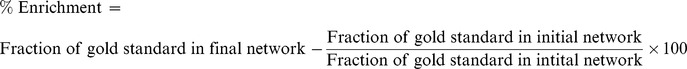
We observed strong enrichment for gold standard interactions using different random subsets of the yeastract TRN with different sizes. The two plots show the hypergeometric p-value and percentage enrichment of gold standard interactions after running GEMINI. The plots show that the effectiveness of GEMINI depends on the scale of the regulatory network. GEMINI evaluates interactions in the context of other interactions in the network and so its effectiveness will depend on the size and degree of completeness of the entire network.(TIF)

Figure S5 Assessment of the algorithm's sensitivity to the choice of the growth threshold used to determine lethal/non-lethal predictions. TF knockouts were predicted to be lethal if the respective maximal growth rate prediction of the mutated organism was less than 5% of the wild-type growth rate. The plot shows that the enrichment for gold standard interactions is robust to the choice of the growth thresholds over a reasonable range of values. While we used the values commonly used in the literature (5%), tuning this threshold indicated that higher enrichments could be achieved by varying this parameter. 10% gave the highest enrichment implying that a 10% cut off might be a better threshold for identifying lethal interactions in yeast. In general, we recommend using the default values to avoid over-fitting.
(TIF)

Figure S6 Estimating the value of κ in a data-driven manner by tuning across a range of values. We set κ to be the lowest value above which there is no increase in the number of interactions removed. We obtained a κ = 10 using this strategy.
(TIF)

Figure S7 Assessment of the algorithm's sensitivity to the choice of the kappa parameter. The enrichment for gold standard interactions is robust to the value of kappa chosen for a wide range of values above 10. Note that higher kappa implies greater constraint due to transcriptional regulation.
(TIF)

Figure S8 Bootstrapping of expression data to determine sensitivity of the algorithm's performance (enrichment for gold standard interactions) to gene expression data size and variance. GEMINI was run using random subsets comprising 80% of the expression data. We found strong enrichment in all of the runs, while complete randomization of gene expression removed enrichment. These results suggest that GEMINI is robust to small changes in gene expression data and the array conditions were quite diverse and were sufficiently powered for this analysis.
(TIF)

Figure S9 Alternative approaches to prioritize interactions. The normalized flux approach works as follows: We first estimated the flux difference between the predicted (v1) and expected (v2) flux state. We then normalized the flux differences to have zero mean and unit variance (z-scores). Reactions were then pooled into two groups based on a threshold z, which represents the deviation from the mean flux difference. Interactions that regulate these reactions were then pruned randomly from the first group (higher than the threshold) and then from the second group. The advantage of this approach is that it doesn't rely significantly on the absolute difference between reactions. However this approach introduces a new parameter – the z-score threshold. The plot shows the enrichment for gold standard interactions over a range of z-score thresholds. The fact that we observe strong enrichments using different metrics and thresholds suggest that the systems level constraints are more important than the order in which the different inconsistencies are solved. As mentioned earlier, the flux solutions in FBA have multiple possible states, while the objective function (the growth rate and the transcriptionally constrained reactions) is usually unique.
(TIF)

Figure S10 Changing the threshold for binarizing gene expression data. The binarization threshold is used to binarize the gene expression data for estimating probabilities using PROM. We used the default value used for running PROM (0.33); i.e. genes less than 33^rd^ percentile of the overall expression distribution are considered to be OFF. If the binarization threshold is lowered, only genes with very low expression would be considered as OFF, and we would be unable to quantify interactions accurately. In addition, we may be unable to quantify interactions because some of the genes could be predicted to be ON in all conditions as a result of the low threshold (i.e., lost interactions). Decreasing the threshold to very low values (<0.1) decreases the accuracy of PROM, which leads to less comprehensive prediction. Increasing the threshold above 0.5 decreased the accuracy as well, as it would result in considering genes that are ON as OFF. The ideal region is around 0.3 to 0.4 for running PROM. We performed additional analysis for GEMINI where we tuned our predictions over a range of binarization threshold values. Our accuracy changes with the ability of PROM to accurately predict growth phenotype (Figure S10). We recommend using the default parameter values while running GEMINI.
(TIF)

Table S1 Enriched metabolic pathways in the refined Yeastract network. Table S1a shows the new associations that were obtained by running GEMINI and Supplementary Table S1b shows associations that were removed by running GEMINI. The associations are shown alphabetically (ordered by pathways).
(DOCX)

Table S2 List of 1170 interactions that were predicted by GEMINI to be phenotype-inconsistent in only one of the four conditions (glucose, galactose, glycerol and ethanol). We predicted that these interactions might be true interactions that are conditionally-inactive, and the phenotype inconsistency might have arose due to post transcriptional regulatory mechanisms inactivating these interactions in these conditions. We found that for the top TFs with most interactions in this list were inactivated through phosphorylation, consistent with our predictions.
(DOCX)

Table S3 Analysis of alternate optimal solutions. We compared networks inferred from different flux states by introducing small changes to the expected growth rate. The similarity matrix below shows the network sizes for different growth thresholds (described in the methods section) and their similarity to each other (Table S3a). We also compared refined networks using larger changes in the growth threshold used to find v2. We once again found that while the refined network sizes changed across different thresholds, they were >95% similar to each other. These results indicate that that there is a strong global optimal state for the regulatory network and by perturbing the model and the constraints we still reach very close to the global optima. In terms of network refinement, all these results suggest that there is a core set of regulatory interactions that are removed across different constraints and conditions. (Table S3b).
(DOCX)

## References

[1] Marbach D, Costello JC, Kuffner R, Vega NM, Prill RJ, et al (2012) Wisdom of crowds for robust gene network inference. Nat Methods 9: 796–804. 22796662 10.1038/nmeth.2016 PMC3512113

[2] Marbach D, Roy S, Ay F, Meyer PE, Candeias R, et al (2012) Predictive regulatory models in Drosophila melanogaster by integrative inference of transcriptional networks. Genome Res 22: 1334–1349. 22456606 10.1101/gr.127191.111 PMC3396374

[3] Chandrasekaran S, Ament SA, Eddy JA, Rodriguez-Zas SL, Schatz BR, et al (2011) Behavior-specific changes in transcriptional modules lead to distinct and predictable neurogenomic states. Proc Natl Acad Sci U S A 108: 18020–18025. 21960440 10.1073/pnas.1114093108 PMC3207651

[4] Faith JJ, Hayete B, Thaden JT, Mogno I, Wierzbowski J, et al (2007) Large-scale mapping and validation of Escherichia coli transcriptional regulation from a compendium of expression profiles. PLoS Biol 5: e8. 17214507 10.1371/journal.pbio.0050008 PMC1764438

[5] Bansal M, Belcastro V, Ambesi-Impiombato A, di Bernardo D (2007) How to infer gene networks from expression profiles. Mol Syst Biol 3: 78. 17299415 10.1038/msb4100120 PMC1828749

[6] Friedman N, Linial M, Nachman I, Pe'er D (2000) Using Bayesian networks to analyze expression data. Journal of computational biology 7: 601–620. 11108481 10.1089/106652700750050961

[7] Basso K, Margolin AA, Stolovitzky G, Klein U, Dalla-Favera R, et al (2005) Reverse engineering of regulatory networks in human B cells. Nat Genet 37: 382–390. 15778709 10.1038/ng1532

[8] Bonneau R, Facciotti MT, Reiss DJ, Schmid AK, Pan M, et al (2007) A predictive model for transcriptional control of physiology in a free living cell. Cell 131: 1354–1365. 18160043 10.1016/j.cell.2007.10.053

[9] Babu MM, Lang B, Aravind L (2009) Methods to reconstruct and compare transcriptional regulatory networks. Methods Mol Biol 541: 163–180. 19381525 10.1007/978-1-59745-243-4_8

[10] Roy S, Ernst J, Kharchenko PV, Kheradpour P, Negre N, et al (2010) Identification of functional elements and regulatory circuits by Drosophila modENCODE. Science 330: 1787–1797. 21177974 10.1126/science.1198374 PMC3192495

[11] Harbison CT, Gordon DB, Lee TI, Rinaldi NJ, Macisaac KD, et al (2004) Transcriptional regulatory code of a eukaryotic genome. Nature 431: 99–104. 15343339 10.1038/nature02800 PMC3006441

[12] Rodionov DA (2007) Comparative genomic reconstruction of transcriptional regulatory networks in bacteria. Chem Rev 107: 3467–3497. 17636889 10.1021/cr068309+ PMC2643304

[13] Fendt SM, Oliveira AP, Christen S, Picotti P, Dechant RC, et al (2010) Unraveling condition-dependent networks of transcription factors that control metabolic pathway activity in yeast. Mol Syst Biol 6: 432. 21119627 10.1038/msb.2010.91 PMC3010106

[14] Bonneau R (2008) Learning biological networks: from modules to dynamics. Nat Chem Biol 4: 658–664. 18936750 10.1038/nchembio.122

[15] Hu Z, Killion PJ, Iyer VR (2007) Genetic reconstruction of a functional transcriptional regulatory network. Nat Genet 39: 683–687. 17417638 10.1038/ng2012

[16] Lewis NE, Nagarajan H, Palsson BO (2012) Constraining the metabolic genotype-phenotype relationship using a phylogeny of in silico methods. Nat Rev Microbiol 10: 291–305. 22367118 10.1038/nrmicro2737 PMC3536058

[17] Price ND, Reed JL, Palsson BO (2004) Genome-scale models of microbial cells: evaluating the consequences of constraints. Nat Rev Microbiol 2: 886–897. 15494745 10.1038/nrmicro1023

[18] Covert MW, Knight EM, Reed JL, Herrgard MJ, Palsson BO (2004) Integrating high-throughput and computational data elucidates bacterial networks. Nature 429: 92–96. 15129285 10.1038/nature02456

[19] Herrgard MJ, Lee BS, Portnoy V, Palsson BO (2006) Integrated analysis of regulatory and metabolic networks reveals novel regulatory mechanisms in Saccharomyces cerevisiae. Genome Res 16: 627–635. 16606697 10.1101/gr.4083206 PMC1457053

[20] Feist AM, Herrgard MJ, Thiele I, Reed JL, Palsson BO (2009) Reconstruction of biochemical networks in microorganisms. Nat Rev Microbiol 7: 129–143. 19116616 10.1038/nrmicro1949 PMC3119670

[21] Kumar VS, Maranas CD (2009) GrowMatch: an automated method for reconciling in silico/in vivo growth predictions. PLoS Comput Biol 5: e1000308. 19282964 10.1371/journal.pcbi.1000308 PMC2645679

[22] Chandrasekaran S, Price ND (2010) Probabilistic integrative modeling of genome-scale metabolic and regulatory networks in Escherichia coli and Mycobacterium tuberculosis. Proc Natl Acad Sci U S A 107: 17845–17850. 20876091 10.1073/pnas.1005139107 PMC2955152

[23] Brenner S (2010) Sequences and consequences. Philos Trans R Soc Lond B Biol Sci 365: 207–212. 20008397 10.1098/rstb.2009.0221 PMC2842711

[24] Barrett CL, Herring CD, Reed JL, Palsson BO (2005) The global transcriptional regulatory network for metabolism in Escherichia coli exhibits few dominant functional states. Proc Natl Acad Sci U S A 102: 19103–19108. 16357206 10.1073/pnas.0505231102 PMC1323155

[25] Lee JM, Gianchandani EP, Eddy JA, Papin JA (2008) Dynamic analysis of integrated signaling, metabolic, and regulatory networks. PLoS Comput Biol 4: e1000086. 18483615 10.1371/journal.pcbi.1000086 PMC2377155

[26] Shlomi T, Cabili MN, Herrgard MJ, Palsson BO, Ruppin E (2008) Network-based prediction of human tissue-specific metabolism. Nat Biotechnol 26: 1003–1010. 18711341 10.1038/nbt.1487

[27] Barua D, Kim J, Reed JL (2010) An automated phenotype-driven approach (GeneForce) for refining metabolic and regulatory models. PLoS Comput Biol 6: e1000970. 21060853 10.1371/journal.pcbi.1000970 PMC2965739

[28] Orth JD, Thiele I, Palsson BØ (2010) What is flux balance analysis? Nature biotechnology 28: 245–248. 10.1038/nbt.1614 PMC3108565 20212490

[29] Mahadevan R, Schilling CH (2003) The effects of alternate optimal solutions in constraint-based genome-scale metabolic models. Metab Eng 5: 264–276. 14642354 10.1016/j.ymben.2003.09.002

[30] Schaffter T, Marbach D, Floreano D (2011) GeneNetWeaver: in silico benchmark generation and performance profiling of network inference methods. Bioinformatics 27: 2263–2270. 21697125 10.1093/bioinformatics/btr373

[31] Abdulrehman D, Monteiro PT, Teixeira MC, Mira NP, Lourenco AB, et al (2011) YEASTRACT: providing a programmatic access to curated transcriptional regulatory associations in Saccharomyces cerevisiae through a web services interface. Nucleic Acids Res 39: D136–140. 20972212 10.1093/nar/gkq964 PMC3013800

[32] Teixeira MC, Monteiro P, Jain P, Tenreiro S, Fernandes AR, et al (2006) The YEASTRACT database: a tool for the analysis of transcription regulatory associations in Saccharomyces cerevisiae. Nucleic Acids Res 34: D446–451. 16381908 10.1093/nar/gkj013 PMC1347376

[33] Zomorrodi AR, Maranas CD (2010) Improving the iMM904 S. cerevisiae metabolic model using essentiality and synthetic lethality data. BMC Syst Biol 4: 178. 21190580 10.1186/1752-0509-4-178 PMC3023687

[34] Faith JJ, Driscoll ME, Fusaro VA, Cosgrove EJ, Hayete B, et al (2008) Many Microbe Microarrays Database: uniformly normalized Affymetrix compendia with structured experimental metadata. Nucleic Acids Res 36: D866–870. 17932051 10.1093/nar/gkm815 PMC2238822

[35] Kuepfer L, Sauer U, Blank LM (2005) Metabolic functions of duplicate genes in Saccharomyces cerevisiae. Genome Res 15: 1421–1430. 16204195 10.1101/gr.3992505 PMC1240085

[36] Oldham MC, Konopka G, Iwamoto K, Langfelder P, Kato T, et al (2008) Functional organization of the transcriptome in human brain. Nat Neurosci 11: 1271–1282. 18849986 10.1038/nn.2207 PMC2756411

[37] Reimand J, Vaquerizas JM, Todd AE, Vilo J, Luscombe NM (2010) Comprehensive reanalysis of transcription factor knockout expression data in Saccharomyces cerevisiae reveals many new targets. Nucleic Acids Res 38: 4768–4777. 20385592 10.1093/nar/gkq232 PMC2919724

[38] Zhu C, Byers KJ, McCord RP, Shi Z, Berger MF, et al (2009) High-resolution DNA-binding specificity analysis of yeast transcription factors. Genome Res 19: 556–566. 19158363 10.1101/gr.090233.108 PMC2665775

[39] MacIsaac KD, Wang T, Gordon DB, Gifford DK, Stormo GD, et al (2006) An improved map of conserved regulatory sites for Saccharomyces cerevisiae. BMC Bioinformatics 7: 113. 16522208 10.1186/1471-2105-7-113 PMC1435934

[40] Yu H, Gerstein M (2006) Genomic analysis of the hierarchical structure of regulatory networks. Proc Natl Acad Sci U S A 103: 14724–14731. 17003135 10.1073/pnas.0508637103 PMC1595419

[41] Mo ML, Palsson BO, Herrgard MJ (2009) Connecting extracellular metabolomic measurements to intracellular flux states in yeast. BMC Syst Biol 3: 37. 19321003 10.1186/1752-0509-3-37 PMC2679711

[42] Bro C, Knudsen S, Regenberg B, Olsson L, Nielsen J (2005) Improvement of galactose uptake in Saccharomyces cerevisiae through overexpression of phosphoglucomutase: example of transcript analysis as a tool in inverse metabolic engineering. Appl Environ Microbiol 71: 6465–6472. 16269670 10.1128/AEM.71.11.6465-6472.2005 PMC1287681

[43] Ideker T, Thorsson V, Ranish JA, Christmas R, Buhler J, et al (2001) Integrated genomic and proteomic analyses of a systematically perturbed metabolic network. Science 292: 929–934. 11340206 10.1126/science.292.5518.929

[44] Fendt SM, Sauer U (2010) Transcriptional regulation of respiration in yeast metabolizing differently repressive carbon substrates. BMC Syst Biol 4: 12. 20167065 10.1186/1752-0509-4-12 PMC2847992

[45] Hashikawa N, Mizukami Y, Imazu H, Sakurai H (2006) Mutated yeast heat shock transcription factor activates transcription independently of hyperphosphorylation. J Biol Chem 281: 3936–3942. 16361698 10.1074/jbc.M510827200

[46] Young ET, Dombek KM, Tachibana C, Ideker T (2003) Multiple pathways are co-regulated by the protein kinase Snf1 and the transcription factors Adr1 and Cat8. J Biol Chem 278: 26146–26158. 12676948 10.1074/jbc.M301981200

[47] Orzechowski Westholm J, Tronnersjo S, Nordberg N, Olsson I, Komorowski J, et al (2012) Gis1 and Rph1 regulate glycerol and acetate metabolism in glucose depleted yeast cells. PLoS One 7: e31577. 22363679 10.1371/journal.pone.0031577 PMC3283669

[48] Henry CS, DeJongh M, Best AA, Frybarger PM, Linsay B, et al (2010) High-throughput generation, optimization and analysis of genome-scale metabolic models. Nat Biotechnol 28: 977–982. 20802497 10.1038/nbt.1672

[49] Karr JR, Sanghvi JC, Macklin DN, Gutschow MV, Jacobs JM, et al (2012) A whole-cell computational model predicts phenotype from genotype. Cell 150: 389–401. 22817898 10.1016/j.cell.2012.05.044 PMC3413483

[50] Shlomi T, Berkman O, Ruppin E (2005) Regulatory on/off minimization of metabolic flux changes after genetic perturbations. Proc Natl Acad Sci U S A 102: 7695–7700. 15897462 10.1073/pnas.0406346102 PMC1140402

[51] Gudmundsson S, Thiele I (2010) Computationally efficient flux variability analysis. BMC Bioinformatics 11: 489. 20920235 10.1186/1471-2105-11-489 PMC2963619

[52] Simeonidis E, Chandrasekaran S, Price N (2013) A Guide to Integrating Transcriptional Regulatory and Metabolic Networks Using PROM (Probabilistic Regulation of Metabolism). In: Alper HS, editor. Systems Metabolic Engineering: Humana Press. pp. 103–112. 10.1007/978-1-62703-299-5_6 23417801

[53] Becker SA, Feist AM, Mo ML, Hannum G, Palsson BO, et al (2007) Quantitative prediction of cellular metabolism with constraint-based models: the COBRA Toolbox. Nat Protoc 2: 727–738. 17406635 10.1038/nprot.2007.99

