# Supplementary figures and images for: Metabolic Constraint-Based Refinement of Transcriptional Regulatory Networks
Source: PLoS Comput Biol. 2013 Dec 5;9(12):e1003370. doi: 10.1371/journal.pcbi.1003370 (PMC3857774; doi:10.1371/journal.pcbi.1003370)

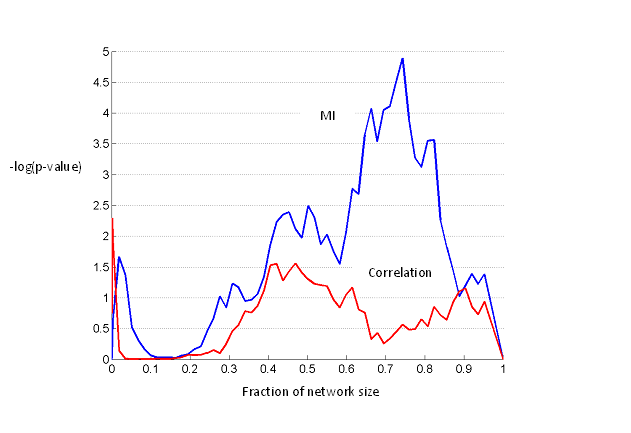

Supplement: Figure S1 — Mutual Information (blue) and correlation (red) tuning across various network sizes. Plots show enrichment (shown as the negative log to the base 10 of the hypergeometric p-value) for direct interactions. The same gene expression data set used for GEMINI (904 arrays in 435 conditions) from the Many-Microbes Microarray Database were used for estimating MI and correlation. Interestingly, we observed that redoing the same analysis using interactions with positive pearson's correlation alone yielded higher enrichments (minimum p-value = 10−10), while interactions with negative pearson's correlation did not lead to any enrichment for direct interactions (minimum p-value = 0.99). (TIF) [file pcbi.1003370.s001.tif]

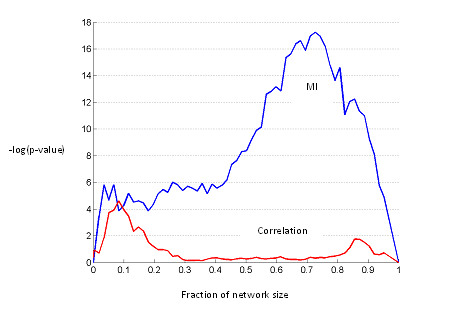

Supplement: Figure S2 — MI (blue) and correlation (red) tuning across various network sizes for indirect interactions. Plots show enrichment (shown as the negative log to the base 10 of the hypergeometric p-value) for indirect interactions. Interestingly, similar to direct interactions, looking at interactions with positive pearson's correlation alone yielded higher enrichments (minimum p-value = 10−16), while interactions with negative pearson's correlation did not lead to any enrichment for indirect interactions (minimum p-value = 0.96). (TIF) [file pcbi.1003370.s002.tif]

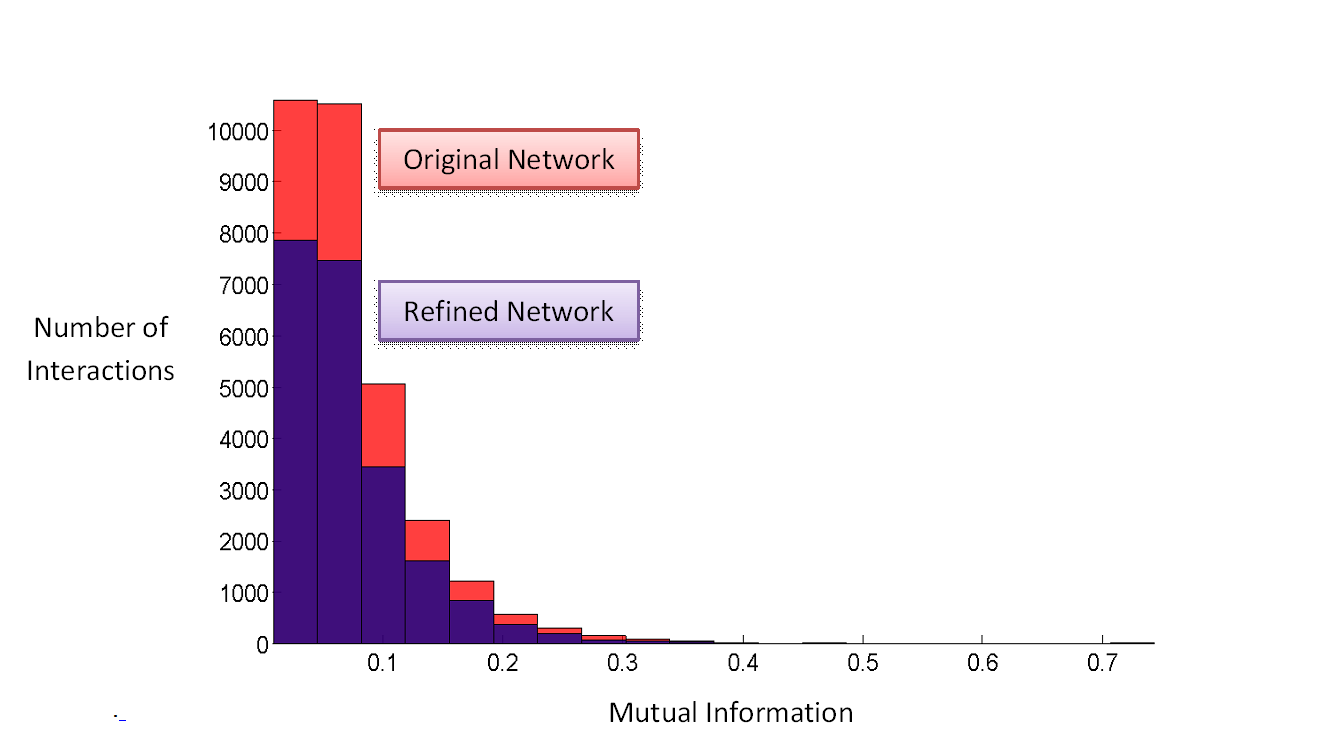

Supplement: Figure S3 — Comparison of the distributions of the MI scores for the original and refined yeastract networks. We found that the interactions retained by GEMINI do not consist only of the lower part of the total MI distribution, except for extremely low MI values close to zero. The pruning of the network by GEMINI is less trivial than simply raising the threshold to select for significant MI scores. The Kolmogorov-Smirnoff test also revealed no significant difference (p-value = 1) between the two MI distributions. (TIF) [file pcbi.1003370.s003.tif]

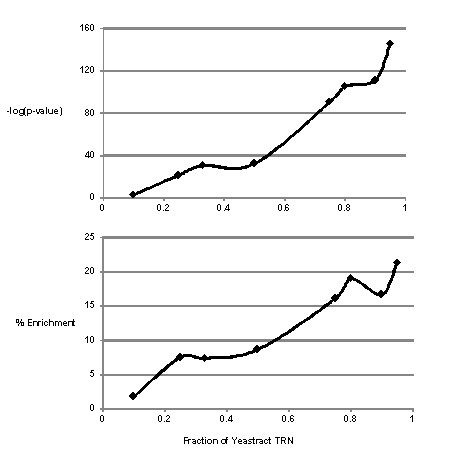

Supplement: Figure S4 — Effect of the size of the input TRN We observed strong enrichment for gold standard interactions using different random subsets of the yeastract TRN with different sizes. The two plots show the hypergeometric p-value and percentage enrichment of gold standard interactions after running GEMINI. The plots show that the effectiveness of GEMINI depends on the scale of the regulatory network. GEMINI evaluates interactions in the context of other interactions in the network and so its effectiveness will depend on the size and degree of completeness of the entire network. (TIF) [file pcbi.1003370.s004.tif]

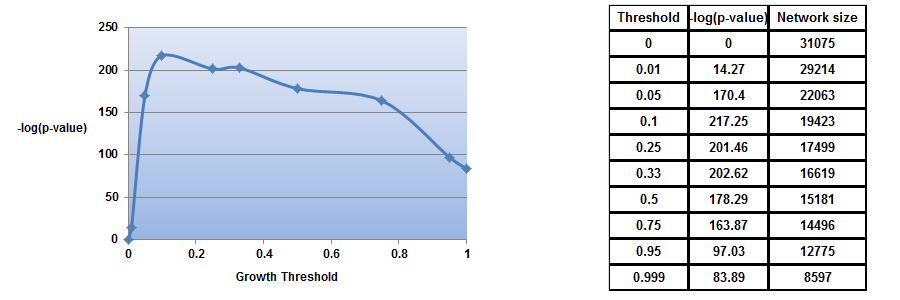

Supplement: Figure S5 — Assessment of the algorithm's sensitivity to the choice of the growth threshold used to determine lethal/non-lethal predictions. TF knockouts were predicted to be lethal if the respective maximal growth rate prediction of the mutated organism was less than 5% of the wild-type growth rate. The plot shows that the enrichment for gold standard interactions is robust to the choice of the growth thresholds over a reasonable range of values. While we used the values commonly used in the literature (5%), tuning this threshold indicated that higher enrichments could be achieved by varying this parameter. 10% gave the highest enrichment implying that a 10% cut off might be a better threshold for identifying lethal interactions in yeast. In general, we recommend using the default values to avoid over-fitting. (TIF) [file pcbi.1003370.s005.tif]

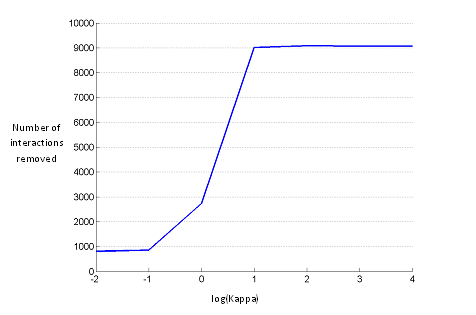

Supplement: Figure S6 — Estimating the value of κ in a data-driven manner by tuning across a range of values. We set κ to be the lowest value above which there is no increase in the number of interactions removed. We obtained a κ = 10 using this strategy. (TIF) [file pcbi.1003370.s006.tif]

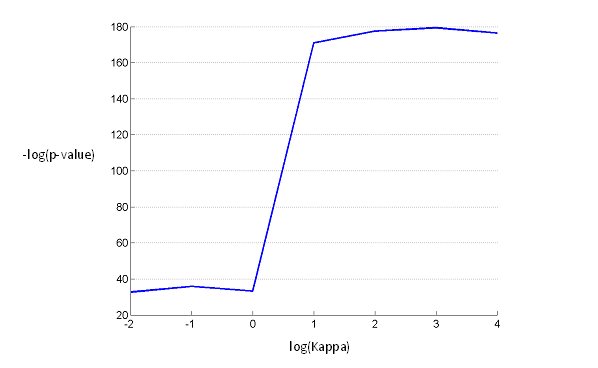

Supplement: Figure S7 — Assessment of the algorithm's sensitivity to the choice of the kappa parameter. The enrichment for gold standard interactions is robust to the value of kappa chosen for a wide range of values above 10. Note that higher kappa implies greater constraint due to transcriptional regulation. (TIF) [file pcbi.1003370.s007.tif]

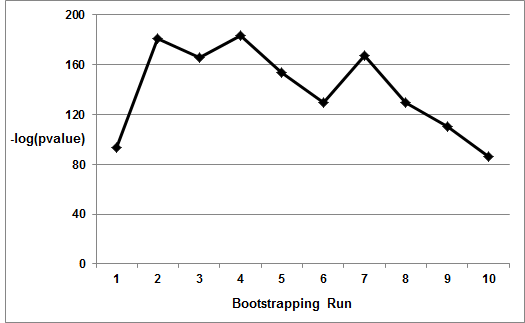

Supplement: Figure S8 — Bootstrapping of expression data to determine sensitivity of the algorithm's performance (enrichment for gold standard interactions) to gene expression data size and variance. GEMINI was run using random subsets comprising 80% of the expression data. We found strong enrichment in all of the runs, while complete randomization of gene expression removed enrichment. These results suggest that GEMINI is robust to small changes in gene expression data and the array conditions were quite diverse and were sufficiently powered for this analysis. (TIF) [file pcbi.1003370.s008.tif]

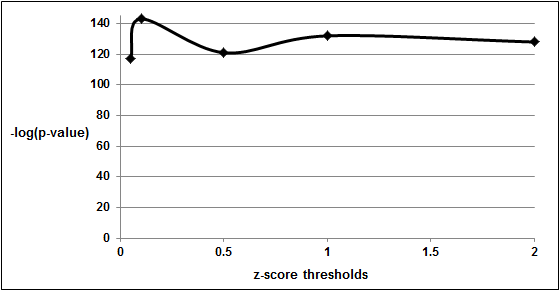

Supplement: Figure S9 — Alternative approaches to prioritize interactions. The normalized flux approach works as follows: We first estimated the flux difference between the predicted (v1) and expected (v2) flux state. We then normalized the flux differences to have zero mean and unit variance (z-scores). Reactions were then pooled into two groups based on a threshold z, which represents the deviation from the mean flux difference. Interactions that regulate these reactions were then pruned randomly from the first group (higher than the threshold) and then from the second group. The advantage of this approach is that it doesn't rely significantly on the absolute difference between reactions. However this approach introduces a new parameter – the z-score threshold. The plot shows the enrichment for gold standard interactions over a range of z-score thresholds. The fact that we observe strong enrichments using different metrics and thresholds suggest that the systems level constraints are more important than the order in which the different inconsistencies are solved. As mentioned earlier, the flux solutions in FBA have multiple possible states, while the objective function (the growth rate and the transcriptionally constrained reactions) is usually unique. (TIF) [file pcbi.1003370.s009.tif]

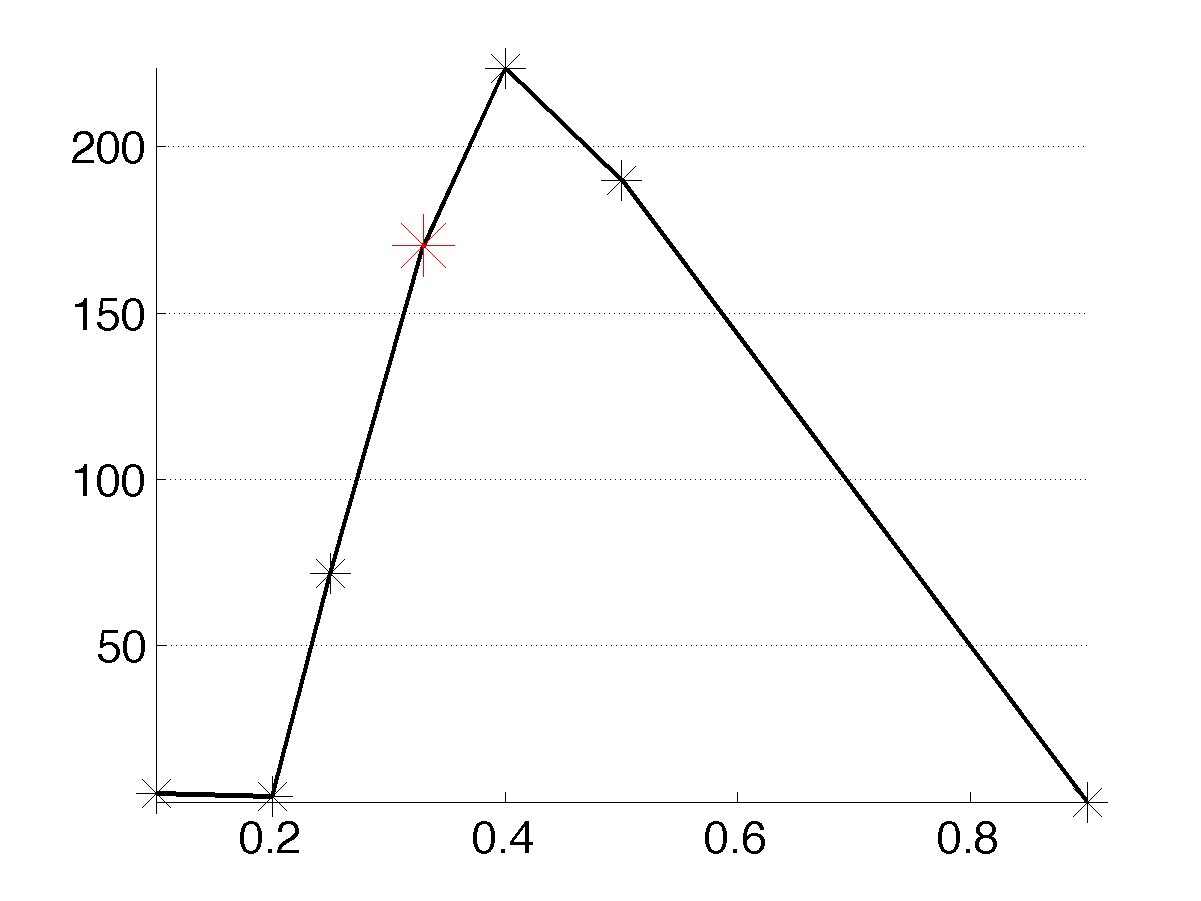

Supplement: Figure S10 — Changing the threshold for binarizing gene expression data. The binarization threshold is used to binarize the gene expression data for estimating probabilities using PROM. We used the default value used for running PROM (0.33); i.e. genes less than 33rd percentile of the overall expression distribution are considered to be OFF. If the binarization threshold is lowered, only genes with very low expression would be considered as OFF, and we would be unable to quantify interactions accurately. In addition, we may be unable to quantify interactions because some of the genes could be predicted to be ON in all conditions as a result of the low threshold (i.e., lost interactions). Decreasing the threshold to very low values (<0.1) decreases the accuracy of PROM, which leads to less comprehensive prediction. Increasing the threshold above 0.5 decreased the accuracy as well, as it would result in considering genes that are ON as OFF. The ideal region is around 0.3 to 0.4 for running PROM. We performed additional analysis for GEMINI where we tuned our predictions over a range of binarization threshold values. Our accuracy changes with the ability of PROM to accurately predict growth phenotype (Figure S10). We recommend using the default parameter values while running GEMINI. (TIF) [file pcbi.1003370.s010.tif]
