# Supplementary material for: Metabolic Constraint-Based Refinement of Transcriptional Regulatory Networks
Source: PLoS Comput Biol. 2013 Dec 5;9(12):e1003370. doi: 10.1371/journal.pcbi.1003370 (PMC3857774; doi:10.1371/journal.pcbi.1003370)
Supplement: Table S1 — Enriched metabolic pathways in the refined Yeastract network. Table S1a shows the new associations that were obtained by running GEMINI and Supplementary Table S1b shows associations that were removed by running GEMINI. The associations are shown alphabetically (ordered by pathways). (DOCX) [file pcbi.1003370.s011.docx]

Supplementary Table S1a: New associations

| SFP1 | Complex Alcohol Metabolism |
| --- | --- |
| HSF1 | Fatty Acid Degradation |
| XBP1 | Glycerolipid Metabolism |
| FKH2 | Glycerolipid Metabolism |
| ASH1 | Glycolysis Gluconeogenesis |
| MSN2 | Glycoprotein Metabolism |
| GIS1 | Glycoprotein Metabolism |
| MSN4 | Glycoprotein Metabolism |
| PHO4 | Glycoprotein Metabolism |
| YER130c | Glycoprotein Metabolism |
| RPH1 | Glycoprotein Metabolism |
| YAP1 | NAD Biosynthesis |
| GZF3 | Other Amino Acid Metabolism |
| ADR1 | Pentose Phosphate Pathway |
| HSF1 | Phospholipid Biosynthesis |
| YAP1 | Phospholipid Biosynthesis |
| HSF1 | Porphyrin and Chlorophyll Metabolism |
| RTG3 | Thiamine Metabolism |
| FKH2 | Thiamine Metabolism |
| NRG1 | Thiamine Metabolism |
| FKH1 | Thiamine Metabolism |
| SFP1 | Thiamine Metabolism |
| ASH1 | Transport Extracellular |
| BAS1 | Transport Extracellular |
| FKH1 | Transport Extracellular |
| ADR1 | Transport Extracellular |
| GIS1 | Transport Extracellular |
| FKH2 | Transport Extracellular |
| GLN3 | Transport Extracellular |
| SFP1 | Transport Extracellular |
| PHO4 | Transport Extracellular |
| RTG3 | Transport Extracellular |
| NRG1 | Transport Extracellular |
| MSN2 | Transport Extracellular |
| XBP1 | Transport Extracellular |
| GZF3 | Transport Extracellular |
| MSN4 | Transport Extracellular |
| RPH1 | Transport Extracellular |
| FKH2 | tRNA charging |
| FKH1 | tRNA charging |

Supplementary Table S1b: Associations removed

| SKN7 | Alternate Carbon Metabolism |
| --- | --- |
| CRZ1 | Alternate Carbon Metabolism |
| BAS1 | Arginine and Proline Metabolism |
| GIS1 | Citric Acid Cycle |
| MSN4 | Citric Acid Cycle |
| MSN4 | Fatty Acid Degradation |
| MSN2 | Fatty Acid Degradation |
| YAP1 | Glutamate metabolism |
| GIS1 | Pentose Phosphate Pathway |
| YER130c | Pentose Phosphate Pathway |
| RPH1 | Pentose Phosphate Pathway |
| PHO4 | Pentose Phosphate Pathway |
| MSN4 | Pentose Phosphate Pathway |
| PHO4 | Phospholipid Biosynthesis |
| BAS1 | Purine and Pyrimidine Biosynthesis |
| GIS1 | Pyruvate Metabolism |
| YER130c | Pyruvate Metabolism |
| MSN4 | Pyruvate Metabolism |
| YRR1 | Sphingolipid Metabolism |
| SFP1 | Sterol Metabolism |
| SFP1 | Valine Leucine and Isoleucine Metabolism |
