# Supplementary material for: Metabolic Constraint-Based Refinement of Transcriptional Regulatory Networks
Source: PLoS Comput Biol. 2013 Dec 5;9(12):e1003370. doi: 10.1371/journal.pcbi.1003370 (PMC3857774; doi:10.1371/journal.pcbi.1003370)
Supplement: Table S2 — List of 1170 interactions that were predicted by GEMINI to be phenotype-inconsistent in only one of the four conditions (glucose, galactose, glycerol and ethanol). We predicted that these interactions might be true interactions that are conditionally-inactive, and the phenotype inconsistency might have arose due to post transcriptional regulatory mechanisms inactivating these interactions in these conditions. We found that for the top TFs with most interactions in this list were inactivated through phosphorylation, consistent with our predictions. (DOCX) [file pcbi.1003370.s012.docx]

Supplementary Table 2: List of 1170 interactions that were predicted by GEMINI to be phenotype-inconsistent in only one of the four conditions (glucose, galactose, glycerol and ethanol). We predicted that these interactions might be true interactions that are conditionally-inactive, and the phenotype inconsistency might have arose due to post transcriptional regulatory mechanisms inactivating these interactions in these conditions. We found that for the top TFs with most interactions in this list were inactivated through phosphorylation, consistent with our predictions.

| Regulator | Regulator Symbol | Target | Target Symbol | Condition |  | Regulator | Regulator Symbol | Target | Target Symbol | Condition |
| --- | --- | --- | --- | --- | --- | --- | --- | --- | --- | --- |
| YBL103C | RTG3 | YOR126C | IAH1 | Glucose |  | YKL109W | HAP4 | YBR153W | RIB7 | Galactose |
| YDR096W | GIS1 | YNR041C | COQ2 | Glucose |  | YKL109W | HAP4 | YCL018W | LEU2 | Galactose |
| YDR096W | GIS1 | YOR074C | CDC21 | Glucose |  | YKL109W | HAP4 | YCL040W | GLK1 | Galactose |
| YDR096W | GIS1 | YPL092W | SSU1 | Glucose |  | YKL109W | HAP4 | YDL004W | ATP16 | Galactose |
| YDR096W | GIS1 | YPR033C | HTS1 | Glucose |  | YKL109W | HAP4 | YDL022W | GPD1 | Galactose |
| YDR216W | ADR1 | YNR008W | LRO1 | Glucose |  | YKL109W | HAP4 | YDL067C | COX9 | Galactose |
| YDR216W | ADR1 | YNR012W | URK1 | Glucose |  | YKL109W | HAP4 | YDL168W | SFA1 | Galactose |
| YDR216W | ADR1 | YOL143C | RIB4 | Glucose |  | YKL109W | HAP4 | YDL181W | INH1 | Galactose |
| YDR216W | ADR1 | YPL092W | SSU1 | Glucose |  | YKL109W | HAP4 | YDR007W | TRP1 | Galactose |
| YDR216W | ADR1 | YPR033C | HTS1 | Glucose |  | YKL109W | HAP4 | YDR148C | KGD2 | Galactose |
| YDR216W | ADR1 | YPR140W | TAZ1 | Glucose |  | YKL109W | HAP4 | YDR178W | SDH4 | Galactose |
| YER040W | GLN3 | YPL092W | SSU1 | Glucose |  | YKL109W | HAP4 | YDR226W | ADK1 | Galactose |
| YER169W | RPH1 | Q0085 | ATP6 | Glucose |  | YKL109W | HAP4 | YDR298C | ATP5 | Galactose |
| YER169W | RPH1 | YAL038W | CDC19 | Glucose |  | YKL109W | HAP4 | YDR322C-A | TIM11 | Galactose |
| YER169W | RPH1 | YAL044C | GCV3 | Glucose |  | YKL109W | HAP4 | YDR341C | YDR341c | Galactose |
| YER169W | RPH1 | YAL054C | ACS1 | Glucose |  | YKL109W | HAP4 | YDR354W | TRP4 | Galactose |
| YER169W | RPH1 | YAL060W | BDH1 | Glucose |  | YKL109W | HAP4 | YDR377W | ATP17 | Galactose |
| YER169W | RPH1 | YAR015W | ADE1 | Glucose |  | YKL109W | HAP4 | YDR408C | ADE8 | Galactose |
| YER169W | RPH1 | YBL011W | SCT1 | Glucose |  | YKL109W | HAP4 | YDR502C | SAM2 | Galactose |
| YER169W | RPH1 | YBL013W | FMT1 | Glucose |  | YKL109W | HAP4 | YDR529C | QCR7 | Galactose |
| YER169W | RPH1 | YBL015W | ACH1 | Glucose |  | YKL109W | HAP4 | YEL024W | RIP1 | Galactose |
| YER169W | RPH1 | YBL033C | RIB1 | Glucose |  | YKL109W | HAP4 | YER003C | PMI40 | Galactose |
| YER169W | RPH1 | YBL039C | URA7 | Glucose |  | YKL109W | HAP4 | YER026C | CHO1 | Galactose |
| YER169W | RPH1 | YBL045C | COR1 | Glucose |  | YKL109W | HAP4 | YER052C | HOM3 | Galactose |
| YER169W | RPH1 | YBL064C | PRX1 | Glucose |  | YKL109W | HAP4 | YER055C | HIS1 | Galactose |
| YER169W | RPH1 | YBL098W | BNA4 | Glucose |  | YKL109W | HAP4 | YER069W | ARG5,6 | Galactose |
| YER169W | RPH1 | YBL099W | ATP1 | Glucose |  | YKL109W | HAP4 | YER070W | RNR1 | Galactose |
| YER169W | RPH1 | YBR001C | NTH2 | Glucose |  | YKL109W | HAP4 | YER178W | PDA1 | Galactose |
| YER169W | RPH1 | YBR003W | COQ1 | Glucose |  | YKL109W | HAP4 | YFL018C | LPD1 | Galactose |
| YER169W | RPH1 | YBR018C | GAL7 | Glucose |  | YKL109W | HAP4 | YFL022C | FRS2 | Galactose |
| YER169W | RPH1 | YBR021W | FUR4 | Glucose |  | YKL109W | HAP4 | YFR033C | QCR6 | Galactose |
| YER169W | RPH1 | YBR026C | ETR1 | Glucose |  | YKL109W | HAP4 | YGL001C | ERG26 | Galactose |
| YER169W | RPH1 | YBR035C | PDX3 | Glucose |  | YKL109W | HAP4 | YGL009C | LEU1 | Galactose |
| YER169W | RPH1 | YBR036C | CSG2 | Glucose |  | YKL109W | HAP4 | YGL187C | COX4 | Galactose |
| YER169W | RPH1 | YBR038W | CHS2 | Glucose |  | YKL109W | HAP4 | YGL191W | COX13 | Galactose |
| YER169W | RPH1 | YBR069C | TAT1 | Glucose |  | YKL109W | HAP4 | YGL205W | POX1 | Galactose |
| YER169W | RPH1 | YBR084W | MIS1 | Glucose |  | YKL109W | HAP4 | YGL256W | ADH4 | Galactose |
| YER169W | RPH1 | YBR115C | LYS2 | Glucose |  | YKL109W | HAP4 | YGR175C | ERG1 | Galactose |
| YER169W | RPH1 | YBR117C | TKL2 | Glucose |  | YKL109W | HAP4 | YGR183C | QCR9 | Galactose |
| YER169W | RPH1 | YBR126C | TPS1 | Glucose |  | YKL109W | HAP4 | YGR193C | PDX1 | Galactose |
| YER169W | RPH1 | YBR132C | AGP2 | Glucose |  | YKL109W | HAP4 | YGR240C | PFK1 | Galactose |
| YER169W | RPH1 | YBR145W | ADH5 | Glucose |  | YKL109W | HAP4 | YGR260W | TNA1 | Galactose |
| YER169W | RPH1 | YBR149W | ARA1 | Glucose |  | YKL109W | HAP4 | YHR001W-A | QCR10 | Galactose |
| YER169W | RPH1 | YBR161W | CSH1 | Glucose |  | YKL109W | HAP4 | YHR007C | ERG11 | Galactose |
| YER169W | RPH1 | YBR176W | ECM31 | Glucose |  | YKL109W | HAP4 | YHR019C | DED81 | Galactose |
| YER169W | RPH1 | YBR180W | DTR1 | Glucose |  | YKL109W | HAP4 | YHR051W | COX6 | Galactose |
| YER169W | RPH1 | YBR183W | YPC1 | Glucose |  | YKL109W | HAP4 | YHR072W | ERG7 | Galactose |
| YER169W | RPH1 | YBR192W | RIM2 | Glucose |  | YKL109W | HAP4 | YHR123W | EPT1 | Galactose |
| YER169W | RPH1 | YBR208C | DUR1,2 | Glucose |  | YKL109W | HAP4 | YHR190W | ERG9 | Galactose |
| YER169W | RPH1 | YBR218C | PYC2 | Glucose |  | YKL109W | HAP4 | YIL043C | CBR1 | Galactose |
| YER169W | RPH1 | YBR221C | PDB1 | Glucose |  | YKL109W | HAP4 | YIL111W | COX5b | Galactose |
| YER169W | RPH1 | YBR244W | GPX2 | Glucose |  | YKL109W | HAP4 | YIL125W | KGD1 | Galactose |
| YER169W | RPH1 | YBR256C | RIB5 | Glucose |  | YKL109W | HAP4 | YJL045W | YJL045w | Galactose |
| YER169W | RPH1 | YBR265W | TSC10 | Glucose |  | YKL109W | HAP4 | YJL166W | QCR8 | Galactose |
| YER169W | RPH1 | YBR284W | YBR284w | Glucose |  | YKL109W | HAP4 | YJL167W | ERG20 | Galactose |
| YER169W | RPH1 | YCL004W | PGS1 | Glucose |  | YKL109W | HAP4 | YJL196C | ELO1 | Galactose |
| YER169W | RPH1 | YCL009C | ILV6 | Glucose |  | YKL109W | HAP4 | YJR048W | CYC1 | Galactose |
| YER169W | RPH1 | YCL018W | LEU2 | Glucose |  | YKL109W | HAP4 | YJR073C | OPI3 | Galactose |
| YER169W | RPH1 | YCL025C | AGP1 | Glucose |  | YKL109W | HAP4 | YJR077C | MIR1 | Galactose |
| YER169W | RPH1 | YCL030C | HIS4 | Glucose |  | YKL109W | HAP4 | YJR121W | ATP2 | Galactose |
| YER169W | RPH1 | YCL035C | GRX1 | Glucose |  | YKL109W | HAP4 | YKL016C | ATP7 | Galactose |
| YER169W | RPH1 | YCL040W | GLK1 | Glucose |  | YKL109W | HAP4 | YKL024C | URA6 | Galactose |
| YER169W | RPH1 | YCR005C | CIT2 | Glucose |  | YKL109W | HAP4 | YKL029C | MAE1 | Galactose |
| YER169W | RPH1 | YCR010C | ADY2 | Glucose |  | YKL109W | HAP4 | YKL067W | YNK1 | Galactose |
| YER169W | RPH1 | YCR012W | PGK1 | Glucose |  | YKL109W | HAP4 | YKL085W | MDH1 | Galactose |
| YER169W | RPH1 | YCR024C | SLM5 | Glucose |  | YKL109W | HAP4 | YKL141W | SDH3 | Galactose |
| YER169W | RPH1 | YCR024C-A | PMP1 | Glucose |  | YKL109W | HAP4 | YKL148C | SDH1 | Galactose |
| YER169W | RPH1 | YCR032W | BPH1 | Glucose |  | YKL109W | HAP4 | YKL150W | MCR1 | Galactose |
| YER169W | RPH1 | YCR034W | FEN1 | Glucose |  | YKL109W | HAP4 | YKR009C | FOX2 | Galactose |
| YER169W | RPH1 | YCR048W | ARE1 | Glucose |  | YKL109W | HAP4 | YLL041C | SDH2 | Galactose |
| YER169W | RPH1 | YCR053W | THR4 | Glucose |  | YKL109W | HAP4 | YLR038C | COX12 | Galactose |
| YER169W | RPH1 | YCR073W-A | SOL2 | Glucose |  | YKL109W | HAP4 | YLR043C | TRX1 | Galactose |
| YER169W | RPH1 | YCR083W | TRX3 | Glucose |  | YKL109W | HAP4 | YLR174W | IDP2 | Galactose |
| YER169W | RPH1 | YCR098C | GIT1 | Glucose |  | YKL109W | HAP4 | YLR180W | SAM1 | Galactose |
| YER169W | RPH1 | YDL004W | ATP16 | Glucose |  | YKL109W | HAP4 | YLR209C | PNP1 | Galactose |
| YER169W | RPH1 | YDL022W | GPD1 | Glucose |  | YKL109W | HAP4 | YLR295C | ATP14 | Galactose |
| YER169W | RPH1 | YDL040C | NAT1 | Glucose |  | YKL109W | HAP4 | YLR304C | ACO1 | Galactose |
| YER169W | RPH1 | YDL045C | FAD1 | Glucose |  | YKL109W | HAP4 | YLR372W | SUR4 | Galactose |
| YER169W | RPH1 | YDL052C | SLC1 | Glucose |  | YKL109W | HAP4 | YLR395C | COX8 | Galactose |
| YER169W | RPH1 | YDL066W | IDP1 | Glucose |  | YKL109W | HAP4 | YMR205C | PFK2 | Galactose |
| YER169W | RPH1 | YDL078C | MDH3 | Glucose |  | YKL109W | HAP4 | YMR256C | COX7 | Galactose |
| YER169W | RPH1 | YDL085W | NDE2 | Glucose |  | YKL109W | HAP4 | YMR272C | SCS7 | Galactose |
| YER169W | RPH1 | YDL168W | SFA1 | Glucose |  | YKL109W | HAP4 | YMR303C | ADH2 | Galactose |
| YER169W | RPH1 | YDL171C | GLT1 | Glucose |  | YKL109W | HAP4 | YNL052W | COX5a | Galactose |
| YER169W | RPH1 | YDL178W | DLD2 | Glucose |  | YKL109W | HAP4 | YNL071W | LAT1 | Galactose |
| YER169W | RPH1 | YDL181W | INH1 | Glucose |  | YKL109W | HAP4 | YNL169C | PSD1 | Galactose |
| YER169W | RPH1 | YDL185W | TFP1 | Glucose |  | YKL109W | HAP4 | YNL220W | ADE12 | Galactose |
| YER169W | RPH1 | YDL198C | GGC1 | Glucose |  | YKL109W | HAP4 | YNR050C | LYS9 | Galactose |
| YER169W | RPH1 | YDL205C | HEM3 | Glucose |  | YKL109W | HAP4 | YOL058W | ARG1 | Galactose |
| YER169W | RPH1 | YDL210W | UGA4 | Glucose |  | YKL109W | HAP4 | YOL066C | RIB2 | Galactose |
| YER169W | RPH1 | YDL238C | GUD1 | Glucose |  | YKL109W | HAP4 | YOL086C | ADH1 | Galactose |
| YER169W | RPH1 | YDR001C | NTH1 | Glucose |  | YKL109W | HAP4 | YOL126C | MDH2 | Galactose |
| YER169W | RPH1 | YDR017C | KCS1 | Glucose |  | YKL109W | HAP4 | YOL140W | ARG8 | Galactose |
| YER169W | RPH1 | YDR035W | ARO3 | Glucose |  | YKL109W | HAP4 | YOR065W | CYT1 | Galactose |
| YER169W | RPH1 | YDR044W | HEM13 | Glucose |  | YKL109W | HAP4 | YOR184W | SER1 | Galactose |
| YER169W | RPH1 | YDR050C | TPI1 | Glucose |  | YKL109W | HAP4 | YOR202W | HIS3 | Galactose |
| YER169W | RPH1 | YDR074W | TPS2 | Glucose |  | YKL109W | HAP4 | YOR347C | PYK2 | Galactose |
| YER169W | RPH1 | YDR135C | YCF1 | Glucose |  | YKL109W | HAP4 | YOR375C | GDH1 | Galactose |
| YER169W | RPH1 | YDR148C | KGD2 | Glucose |  | YKL109W | HAP4 | YPL078C | ATP4 | Galactose |
| YER169W | RPH1 | YDR158W | HOM2 | Glucose |  | YKL109W | HAP4 | YPL262W | FUM1 | Galactose |
| YER169W | RPH1 | YDR178W | SDH4 | Glucose |  | YKL109W | HAP4 | YPL271W | ATP15 | Galactose |
| YER169W | RPH1 | YDR204W | COQ4 | Glucose |  | YKL109W | HAP4 | YPR020W | ATP20 | Galactose |
| YER169W | RPH1 | YDR234W | LYS4 | Glucose |  | YKL109W | HAP4 | YPR021C | AGC1 | Galactose |
| YER169W | RPH1 | YDR236C | FMN1 | Glucose |  | YKL109W | HAP4 | YPR060C | ARO7 | Galactose |
| YER169W | RPH1 | YDR294C | DPL1 | Glucose |  | YKL109W | HAP4 | YPR128C | ANT1 | Galactose |
| YER169W | RPH1 | YDR315C | IPK1 | Glucose |  | YKL109W | HAP4 | YPR191W | QCR2 | Galactose |
| YER169W | RPH1 | YDR354W | TRP4 | Glucose |  | YKR099W | BAS1 | YPL087W | YDC1 | Galactose |
| YER169W | RPH1 | YDR377W | ATP17 | Glucose |  | YKR099W | BAS1 | YPL148C | PPT2 | Galactose |
| YER169W | RPH1 | YDR384C | ATO3 | Glucose |  | YKR099W | BAS1 | YPR026W | ATH1 | Galactose |
| YER169W | RPH1 | YDR399W | HPT1 | Glucose |  | YLR014C | PPR1 | YOL143C | RIB4 | Galactose |
| YER169W | RPH1 | YDR402C | DIT2 | Glucose |  | YLR403W | SFP1 | YIL134W | FLX1 | Galactose |
| YER169W | RPH1 | YDR403W | DIT1 | Glucose |  | YLR403W | SFP1 | YPL092W | SSU1 | Galactose |
| YER169W | RPH1 | YDR408C | ADE8 | Glucose |  | YLR403W | SFP1 | YPR033C | HTS1 | Galactose |
| YER169W | RPH1 | YDR453C | TSA2 | Glucose |  | YML007W | YAP1 | YDR297W | SUR2 | Galactose |
| YER169W | RPH1 | YDR481C | PHO8 | Glucose |  | YML007W | YAP1 | YER170W | ADK2 | Galactose |
| YER169W | RPH1 | YDR487C | RIB3 | Glucose |  | YML007W | YAP1 | YFR025C | HIS2 | Galactose |
| YER169W | RPH1 | YDR513W | GRX2 | Glucose |  | YML007W | YAP1 | YFR047C | BNA6 | Galactose |
| YER169W | RPH1 | YDR530C | APA2 | Glucose |  | YML007W | YAP1 | YGR170W | PSD2 | Galactose |
| YER169W | RPH1 | YDR531W | CAB1 | Glucose |  | YML007W | YAP1 | YHR019C | DED81 | Galactose |
| YER169W | RPH1 | YDR536W | STL1 | Glucose |  | YML007W | YAP1 | YHR144C | DCD1 | Galactose |
| YER169W | RPH1 | YEL011W | GLC3 | Glucose |  | YML007W | YAP1 | YJR133W | XPT1 | Galactose |
| YER169W | RPH1 | YEL017C-A | PMP2 | Glucose |  | YML007W | YAP1 | YLR089C | ALT1 | Galactose |
| YER169W | RPH1 | YEL038W | UTR4 | Glucose |  | YML007W | YAP1 | YMR241W | YHM2 | Galactose |
| YER169W | RPH1 | YEL039C | CYC7 | Glucose |  | YML007W | YAP1 | YOL059W | GPD2 | Galactose |
| YER169W | RPH1 | YEL046C | GLY1 | Glucose |  | YML007W | YAP1 | YOR209C | NPT1 | Galactose |
| YER169W | RPH1 | YEL047C | YEL047c | Glucose |  | YML007W | YAP1 | YPR026W | ATH1 | Galactose |
| YER169W | RPH1 | YEL058W | PCM1 | Glucose |  | YML051W | GAL80 | YBR019C | GAL10 | Galactose |
| YER169W | RPH1 | YER052C | HOM3 | Glucose |  | YML051W | GAL80 | YBR020W | GAL1 | Galactose |
| YER169W | RPH1 | YER053C | PIC2 | Glucose |  | YMR016C | SOK2 | YAL054C | ACS1 | Galactose |
| YER169W | RPH1 | YER062C | HOR2 | Glucose |  | YMR016C | SOK2 | YAL062W | GDH3 | Galactose |
| YER169W | RPH1 | YER069W | ARG5,6 | Glucose |  | YMR016C | SOK2 | YAR035W | YAT1 | Galactose |
| YER169W | RPH1 | YER073W | ALD5 | Glucose |  | YMR016C | SOK2 | YBL015W | ACH1 | Galactose |
| YER169W | RPH1 | YER086W | ILV1 | Glucose |  | YMR016C | SOK2 | YBL030C | PET9 | Galactose |
| YER169W | RPH1 | YER090W | TRP2 | Glucose |  | YMR016C | SOK2 | YBL045C | COR1 | Galactose |
| YER169W | RPH1 | YER119C | AVT6 | Glucose |  | YMR016C | SOK2 | YBR019C | GAL10 | Galactose |
| YER169W | RPH1 | YER178W | PDA1 | Glucose |  | YMR016C | SOK2 | YBR020W | GAL1 | Galactose |
| YER169W | RPH1 | YER183C | FAU1 | Glucose |  | YMR016C | SOK2 | YBR085W | AAC3 | Galactose |
| YER169W | RPH1 | YFL017C | GNA1 | Glucose |  | YMR016C | SOK2 | YBR126C | TPS1 | Galactose |
| YER169W | RPH1 | YFL018C | LPD1 | Glucose |  | YMR016C | SOK2 | YBR132C | AGP2 | Galactose |
| YER169W | RPH1 | YFL022C | FRS2 | Glucose |  | YMR016C | SOK2 | YBR296C | PHO89 | Galactose |
| YER169W | RPH1 | YFL030W | AGX1 | Glucose |  | YMR016C | SOK2 | YCL009C | ILV6 | Galactose |
| YER169W | RPH1 | YFL055W | AGP3 | Glucose |  | YMR016C | SOK2 | YCL030C | HIS4 | Galactose |
| YER169W | RPH1 | YFR015C | GSY1 | Glucose |  | YMR016C | SOK2 | YCL064C | CHA1 | Galactose |
| YER169W | RPH1 | YFR019W | FAB1 | Glucose |  | YMR016C | SOK2 | YCR005C | CIT2 | Galactose |
| YER169W | RPH1 | YFR033C | QCR6 | Glucose |  | YMR016C | SOK2 | YCR037C | PHO87 | Galactose |
| YER169W | RPH1 | YGL008C | PMA1 | Glucose |  | YMR016C | SOK2 | YCR053W | THR4 | Galactose |
| YER169W | RPH1 | YGL026C | TRP5 | Glucose |  | YMR016C | SOK2 | YCR098C | GIT1 | Galactose |
| YER169W | RPH1 | YGL037C | PNC1 | Glucose |  | YMR016C | SOK2 | YDL022W | GPD1 | Galactose |
| YER169W | RPH1 | YGL062W | PYC1 | Glucose |  | YMR016C | SOK2 | YDL055C | PSA1 | Galactose |
| YER169W | RPH1 | YGL148W | ARO2 | Glucose |  | YMR016C | SOK2 | YDL078C | MDH3 | Galactose |
| YER169W | RPH1 | YGL154C | LYS5 | Glucose |  | YMR016C | SOK2 | YDR074W | TPS2 | Galactose |
| YER169W | RPH1 | YGL184C | STR3 | Glucose |  | YMR016C | SOK2 | YDR127W | ARO1 | Galactose |
| YER169W | RPH1 | YGL202W | ARO8 | Glucose |  | YMR016C | SOK2 | YDR226W | ADK1 | Galactose |
| YER169W | RPH1 | YGL205W | POX1 | Glucose |  | YMR016C | SOK2 | YDR234W | LYS4 | Galactose |
| YER169W | RPH1 | YGL225W | VRG4 | Glucose |  | YMR016C | SOK2 | YDR380W | ARO10 | Galactose |
| YER169W | RPH1 | YGL234W | ADE5,7 | Glucose |  | YMR016C | SOK2 | YDR384C | ATO3 | Galactose |
| YER169W | RPH1 | YGL248W | PDE1 | Glucose |  | YMR016C | SOK2 | YDR454C | GUK1 | Galactose |
| YER169W | RPH1 | YGL256W | ADH4 | Glucose |  | YMR016C | SOK2 | YEL021W | URA3 | Galactose |
| YER169W | RPH1 | YGR020C | VMA7 | Glucose |  | YMR016C | SOK2 | YEL046C | GLY1 | Galactose |
| YER169W | RPH1 | YGR043C | NQM1 | Glucose |  | YMR016C | SOK2 | YER024W | YAT2 | Galactose |
| YER169W | RPH1 | YGR061C | ADE6 | Glucose |  | YMR016C | SOK2 | YER055C | HIS1 | Galactose |
| YER169W | RPH1 | YGR088W | CTT1 | Glucose |  | YMR016C | SOK2 | YER062C | HOR2 | Galactose |
| YER169W | RPH1 | YGR096W | TPC1 | Glucose |  | YMR016C | SOK2 | YER069W | ARG5,6 | Galactose |
| YER169W | RPH1 | YGR138C | TPO2 | Glucose |  | YMR016C | SOK2 | YFR033C | QCR6 | Galactose |
| YER169W | RPH1 | YGR143W | SKN1 | Glucose |  | YMR016C | SOK2 | YGL009C | LEU1 | Galactose |
| YER169W | RPH1 | YGR155W | CYS4 | Glucose |  | YMR016C | SOK2 | YGL026C | TRP5 | Galactose |
| YER169W | RPH1 | YGR157W | CHO2 | Glucose |  | YMR016C | SOK2 | YGL037C | PNC1 | Galactose |
| YER169W | RPH1 | YGR170W | PSD2 | Glucose |  | YMR016C | SOK2 | YGL125W | MET13 | Galactose |
| YER169W | RPH1 | YGR171C | MSM1 | Glucose |  | YMR016C | SOK2 | YGL205W | POX1 | Galactose |
| YER169W | RPH1 | YGR180C | RNR4 | Glucose |  | YMR016C | SOK2 | YGR060W | ERG25 | Galactose |
| YER169W | RPH1 | YGR183C | QCR9 | Glucose |  | YMR016C | SOK2 | YGR087C | PDC6 | Galactose |
| YER169W | RPH1 | YGR185C | TYS1 | Glucose |  | YMR016C | SOK2 | YGR088W | CTT1 | Galactose |
| YER169W | RPH1 | YGR191W | HIP1 | Glucose |  | YMR016C | SOK2 | YGR121C | MEP1 | Galactose |
| YER169W | RPH1 | YGR193C | PDX1 | Glucose |  | YMR016C | SOK2 | YGR240C | PFK1 | Galactose |
| YER169W | RPH1 | YGR194C | XKS1 | Glucose |  | YMR016C | SOK2 | YGR244C | LSC2 | Galactose |
| YER169W | RPH1 | YGR202C | PCT1 | Glucose |  | YMR016C | SOK2 | YHR002W | LEU5 | Galactose |
| YER169W | RPH1 | YGR209C | TRX2 | Glucose |  | YMR016C | SOK2 | YHR007C | ERG11 | Galactose |
| YER169W | RPH1 | YGR244C | LSC2 | Glucose |  | YMR016C | SOK2 | YHR018C | ARG4 | Galactose |
| YER169W | RPH1 | YGR247W | CPD1 | Glucose |  | YMR016C | SOK2 | YHR190W | ERG9 | Galactose |
| YER169W | RPH1 | YGR248W | SOL4 | Glucose |  | YMR016C | SOK2 | YHR208W | BAT1 | Galactose |
| YER169W | RPH1 | YGR255C | COQ6 | Glucose |  | YMR016C | SOK2 | YIL013C | PDR11 | Galactose |
| YER169W | RPH1 | YGR256W | GND2 | Glucose |  | YMR016C | SOK2 | YIL053W | RHR2 | Galactose |
| YER169W | RPH1 | YGR260W | TNA1 | Glucose |  | YMR016C | SOK2 | YIL094C | LYS12 | Galactose |
| YER169W | RPH1 | YGR264C | MES1 | Glucose |  | YMR016C | SOK2 | YIL116W | HIS5 | Galactose |
| YER169W | RPH1 | YGR267C | FOL2 | Glucose |  | YMR016C | SOK2 | YIL155C | GUT2 | Galactose |
| YER169W | RPH1 | YGR286C | BIO2 | Glucose |  | YMR016C | SOK2 | YIR034C | LYS1 | Galactose |
| YER169W | RPH1 | YHL003C | LAG1 | Glucose |  | YMR016C | SOK2 | YJL026W | RNR2 | Galactose |
| YER169W | RPH1 | YHL016C | DUR3 | Glucose |  | YMR016C | SOK2 | YJL088W | ARG3 | Galactose |
| YER169W | RPH1 | YHL032C | GUT1 | Glucose |  | YMR016C | SOK2 | YJL198W | PHO90 | Galactose |
| YER169W | RPH1 | YHR001W-A | QCR10 | Glucose |  | YMR016C | SOK2 | YJR010W | MET3 | Galactose |
| YER169W | RPH1 | YHR002W | LEU5 | Glucose |  | YMR016C | SOK2 | YJR016C | ILV3 | Galactose |
| YER169W | RPH1 | YHR013C | ARD1 | Glucose |  | YMR016C | SOK2 | YJR148W | BAT2 | Galactose |
| YER169W | RPH1 | YHR018C | ARG4 | Glucose |  | YMR016C | SOK2 | YKL001C | MET14 | Galactose |
| YER169W | RPH1 | YHR019C | DED81 | Glucose |  | YMR016C | SOK2 | YKL029C | MAE1 | Galactose |
| YER169W | RPH1 | YHR025W | THR1 | Glucose |  | YMR016C | SOK2 | YKL182W | FAS1 | Galactose |
| YER169W | RPH1 | YHR026W | PPA1 | Glucose |  | YMR016C | SOK2 | YKL218C | SRY1 | Galactose |
| YER169W | RPH1 | YHR042W | NCP1 | Glucose |  | YMR016C | SOK2 | YKR009C | FOX2 | Galactose |
| YER169W | RPH1 | YHR072W | ERG7 | Glucose |  | YMR016C | SOK2 | YLL028W | TPO1 | Galactose |
| YER169W | RPH1 | YHR074W | QNS1 | Glucose |  | YMR016C | SOK2 | YLL052C | AQY2 | Galactose |
| YER169W | RPH1 | YHR104W | GRE3 | Glucose |  | YMR016C | SOK2 | YLR044C | PDC1 | Galactose |
| YER169W | RPH1 | YHR106W | TRR2 | Glucose |  | YMR016C | SOK2 | YLR056W | ERG3 | Galactose |
| YER169W | RPH1 | YHR137W | ARO9 | Glucose |  | YMR016C | SOK2 | YLR058C | SHM2 | Galactose |
| YER169W | RPH1 | YHR163W | SOL3 | Glucose |  | YMR016C | SOK2 | YLR089C | ALT1 | Galactose |
| YER169W | RPH1 | YHR183W | GND1 | Glucose |  | YMR016C | SOK2 | YLR134W | PDC5 | Galactose |
| YER169W | RPH1 | YHR190W | ERG9 | Glucose |  | YMR016C | SOK2 | YLR295C | ATP14 | Galactose |
| YER169W | RPH1 | YIL013C | PDR11 | Glucose |  | YMR016C | SOK2 | YLR355C | ILV5 | Galactose |
| YER169W | RPH1 | YIL053W | RHR2 | Glucose |  | YMR016C | SOK2 | YML004C | GLO1 | Galactose |
| YER169W | RPH1 | YIL066C | RNR3 | Glucose |  | YMR016C | SOK2 | YML042W | CAT2 | Galactose |
| YER169W | RPH1 | YIL099W | SGA1 | Glucose |  | YMR016C | SOK2 | YML100W | TSL1 | Galactose |
| YER169W | RPH1 | YIL111W | COX5b | Glucose |  | YMR016C | SOK2 | YML123C | PHO84 | Galactose |
| YER169W | RPH1 | YIL124W | AYR1 | Glucose |  | YMR016C | SOK2 | YMR015C | ERG5 | Galactose |
| YER169W | RPH1 | YIL125W | KGD1 | Glucose |  | YMR016C | SOK2 | YMR056C | AAC1 | Galactose |
| YER169W | RPH1 | YIL155C | GUT2 | Glucose |  | YMR016C | SOK2 | YMR062C | ARG7 | Galactose |
| YER169W | RPH1 | YIL162W | SUC2 | Glucose |  | YMR016C | SOK2 | YMR205C | PFK2 | Galactose |
| YER169W | RPH1 | YIR027C | DAL1 | Glucose |  | YMR016C | SOK2 | YNL142W | MEP2 | Galactose |
| YER169W | RPH1 | YIR028W | DAL4 | Glucose |  | YMR016C | SOK2 | YNL277W | MET2 | Galactose |
| YER169W | RPH1 | YIR032C | DAL3 | Glucose |  | YMR016C | SOK2 | YNR001C | CIT1 | Galactose |
| YER169W | RPH1 | YIR034C | LYS1 | Glucose |  | YMR016C | SOK2 | YNR013C | PHO91 | Galactose |
| YER169W | RPH1 | YIR037W | HYR1 | Glucose |  | YMR016C | SOK2 | YNR016C | ACC1 | Galactose |
| YER169W | RPH1 | YJL005W | CYR1 | Glucose |  | YMR016C | SOK2 | YNR050C | LYS9 | Galactose |
| YER169W | RPH1 | YJL026W | RNR2 | Glucose |  | YMR016C | SOK2 | YOL058W | ARG1 | Galactose |
| YER169W | RPH1 | YJL060W | BNA3 | Glucose |  | YMR016C | SOK2 | YOR011W | AUS1 | Galactose |
| YER169W | RPH1 | YJL068C | YJL068c | Glucose |  | YMR016C | SOK2 | YOR095C | RKI1 | Galactose |
| YER169W | RPH1 | YJL070C | YJL070c | Glucose |  | YMR016C | SOK2 | YOR273C | TPO4 | Galactose |
| YER169W | RPH1 | YJL101C | GSH1 | Glucose |  | YMR016C | SOK2 | YOR375C | GDH1 | Galactose |
| YER169W | RPH1 | YJL137C | GLG2 | Glucose |  | YMR016C | SOK2 | YPL023C | MET12 | Galactose |
| YER169W | RPH1 | YJL153C | INO1 | Glucose |  | YMR016C | SOK2 | YPL061W | ALD6 | Galactose |
| YER169W | RPH1 | YJL155C | FBP26 | Glucose |  | YMR016C | SOK2 | YPL147W | PXA1 | Galactose |
| YER169W | RPH1 | YJL167W | ERG20 | Glucose |  | YMR016C | SOK2 | YPR001W | CIT3 | Galactose |
| YER169W | RPH1 | YJL212C | OPT1 | Glucose |  | YMR016C | SOK2 | YPR060C | ARO7 | Galactose |
| YER169W | RPH1 | YJR001W | AVT1 | Glucose |  | YMR016C | SOK2 | YPR138C | MEP3 | Galactose |
| YER169W | RPH1 | YJR010W | MET3 | Glucose |  | YMR016C | SOK2 | YPR192W | AQY1 | Galactose |
| YER169W | RPH1 | YJR066W | TOR1 | Glucose |  | YMR035W | IMP2 | YBR018C | GAL7 | Galactose |
| YER169W | RPH1 | YJR073C | OPI3 | Glucose |  | YMR035W | IMP2 | YBR020W | GAL1 | Galactose |
| YER169W | RPH1 | YJR103W | URA8 | Glucose |  | YMR037C | MSN2 | YOR202W | HIS3 | Galactose |
| YER169W | RPH1 | YJR105W | ADO1 | Glucose |  | YNL027W | CRZ1 | YOR209C | NPT1 | Galactose |
| YER169W | RPH1 | YJR121W | ATP2 | Glucose |  | YNL068C | FKH2 | YOR168W | GLN4 | Galactose |
| YER169W | RPH1 | YJR148W | BAT2 | Glucose |  | YNL068C | FKH2 | YOR335C | ALA1 | Galactose |
| YER169W | RPH1 | YJR153W | PGU1 | Glucose |  | YOR162C | YRR1 | YPR033C | HTS1 | Galactose |
| YER169W | RPH1 | YKL008C | LAC1 | Glucose |  | YOR363C | PIP2 | YBR019C | GAL10 | Galactose |
| YER169W | RPH1 | YKL024C | URA6 | Glucose |  | YOR363C | PIP2 | YDL078C | MDH3 | Galactose |
| YER169W | RPH1 | YKL026C | GPX1 | Glucose |  | YOR363C | PIP2 | YDR074W | TPS2 | Galactose |
| YER169W | RPH1 | YKL029C | MAE1 | Glucose |  | YOR363C | PIP2 | YDR256C | CTA1 | Galactose |
| YER169W | RPH1 | YKL035W | UGP1 | Glucose |  | YOR363C | PIP2 | YDR297W | SUR2 | Galactose |
| YER169W | RPH1 | YKL067W | YNK1 | Glucose |  | YOR363C | PIP2 | YER015W | FAA2 | Galactose |
| YER169W | RPH1 | YKL085W | MDH1 | Glucose |  | YOR363C | PIP2 | YGL205W | POX1 | Galactose |
| YER169W | RPH1 | YKL120W | OAC1 | Glucose |  | YOR363C | PIP2 | YGR088W | CTT1 | Galactose |
| YER169W | RPH1 | YKL127W | PGM1 | Glucose |  | YOR363C | PIP2 | YIL160C | POT1 | Galactose |
| YER169W | RPH1 | YKL148C | SDH1 | Glucose |  | YOR363C | PIP2 | YJL026W | RNR2 | Galactose |
| YER169W | RPH1 | YKL150W | MCR1 | Glucose |  | YOR363C | PIP2 | YJL153C | INO1 | Galactose |
| YER169W | RPH1 | YKL174C | TPO5 | Glucose |  | YOR363C | PIP2 | YKL188C | PXA2 | Galactose |
| YER169W | RPH1 | YKL182W | FAS1 | Glucose |  | YOR363C | PIP2 | YKR009C | FOX2 | Galactose |
| YER169W | RPH1 | YKL184W | SPE1 | Glucose |  | YOR363C | PIP2 | YLR058C | SHM2 | Galactose |
| YER169W | RPH1 | YKL192C | ACP1 | Glucose |  | YOR363C | PIP2 | YLR174W | IDP2 | Galactose |
| YER169W | RPH1 | YKL203C | TOR2 | Glucose |  | YOR363C | PIP2 | YLR284C | ECI1 | Galactose |
| YER169W | RPH1 | YKL216W | URA1 | Glucose |  | YOR363C | PIP2 | YLR355C | ILV5 | Galactose |
| YER169W | RPH1 | YKL217W | JEN1 | Glucose |  | YOR363C | PIP2 | YML042W | CAT2 | Galactose |
| YER169W | RPH1 | YKR009C | FOX2 | Glucose |  | YOR363C | PIP2 | YML100W | TSL1 | Galactose |
| YER169W | RPH1 | YKR031C | SPO14 | Glucose |  | YOR363C | PIP2 | YMR189W | GCV2 | Galactose |
| YER169W | RPH1 | YKR039W | GAP1 | Glucose |  | YOR363C | PIP2 | YMR217W | GUA1 | Galactose |
| YER169W | RPH1 | YKR066C | CCP1 | Glucose |  | YOR363C | PIP2 | YMR272C | SCS7 | Galactose |
| YER169W | RPH1 | YKR067W | GPT2 | Glucose |  | YOR363C | PIP2 | YNL009W | IDP3 | Galactose |
| YER169W | RPH1 | YKR093W | PTR2 | Glucose |  | YOR363C | PIP2 | YNR016C | ACC1 | Galactose |
| YER169W | RPH1 | YKR097W | PCK1 | Glucose |  | YOR363C | PIP2 | YNR050C | LYS9 | Galactose |
| YER169W | RPH1 | YLL018C | DPS1 | Glucose |  | YOR363C | PIP2 | YOL064C | MET22 | Galactose |
| YER169W | RPH1 | YLL028W | TPO1 | Glucose |  | YOR363C | PIP2 | YOL126C | MDH2 | Galactose |
| YER169W | RPH1 | YLL041C | SDH2 | Glucose |  | YOR363C | PIP2 | YOL140W | ARG8 | Galactose |
| YER169W | RPH1 | YLL043W | FPS1 | Glucose |  | YOR363C | PIP2 | YOR180C | DCI1 | Galactose |
| YER169W | RPH1 | YLL048C | YBT1 | Glucose |  | YOR363C | PIP2 | YOR184W | SER1 | Galactose |
| YER169W | RPH1 | YLL052C | AQY2 | Glucose |  | YOR363C | PIP2 | YPL061W | ALD6 | Galactose |
| YER169W | RPH1 | YLL057C | JLP1 | Glucose |  | YOR363C | PIP2 | YPL147W | PXA1 | Galactose |
| YER169W | RPH1 | YLR028C | ADE16 | Glucose |  | YOR363C | PIP2 | YPR128C | ANT1 | Galactose |
| YER169W | RPH1 | YLR056W | ERG3 | Glucose |  | YPL038W | MET31 | YAL012W | CYS3 | Galactose |
| YER169W | RPH1 | YLR070C | XYL2 | Glucose |  | YPL038W | MET31 | YBR020W | GAL1 | Galactose |
| YER169W | RPH1 | YLR089C | ALT1 | Glucose |  | YPL038W | MET31 | YBR115C | LYS2 | Galactose |
| YER169W | RPH1 | YLR100W | ERG27 | Glucose |  | YPL038W | MET31 | YDR050C | TPI1 | Galactose |
| YER169W | RPH1 | YLR109W | AHP1 | Glucose |  | YPL038W | MET31 | YDR454C | GUK1 | Galactose |
| YER169W | RPH1 | YLR133W | CKI1 | Glucose |  | YPL038W | MET31 | YDR502C | SAM2 | Galactose |
| YER169W | RPH1 | YLR138W | NHA1 | Glucose |  | YPL038W | MET31 | YER069W | ARG5,6 | Galactose |
| YER169W | RPH1 | YLR146C | SPE4 | Glucose |  | YPL038W | MET31 | YER091C | MET6 | Galactose |
| YER169W | RPH1 | YLR151C | PCD1 | Glucose |  | YPL038W | MET31 | YGL191W | COX13 | Galactose |
| YER169W | RPH1 | YLR153C | ACS2 | Glucose |  | YPL038W | MET31 | YGR155W | CYS4 | Galactose |
| YER169W | RPH1 | YLR172C | DPH5 | Glucose |  | YPL038W | MET31 | YGR204W | ADE3 | Galactose |
| YER169W | RPH1 | YLR189C | ATG26 | Glucose |  | YPL038W | MET31 | YGR260W | TNA1 | Galactose |
| YER169W | RPH1 | YLR201C | COQ9 | Glucose |  | YPL038W | MET31 | YHR025W | THR1 | Galactose |
| YER169W | RPH1 | YLR240W | VPS34 | Glucose |  | YPL038W | MET31 | YJR010W | MET3 | Galactose |
| YER169W | RPH1 | YLR245C | CDD1 | Glucose |  | YPL038W | MET31 | YKL001C | MET14 | Galactose |
| YER169W | RPH1 | YLR258W | GSY2 | Glucose |  | YPL038W | MET31 | YKL216W | URA1 | Galactose |
| YER169W | RPH1 | YLR284C | ECI1 | Glucose |  | YPL038W | MET31 | YLR027C | AAT2 | Galactose |
| YER169W | RPH1 | YLR285W | NNT1 | Glucose |  | YPL038W | MET31 | YLR174W | IDP2 | Galactose |
| YER169W | RPH1 | YLR295C | ATP14 | Glucose |  | YPL038W | MET31 | YLR180W | SAM1 | Galactose |
| YER169W | RPH1 | YLR299W | ECM38 | Glucose |  | YPL038W | MET31 | YLR303W | MET17 | Galactose |
| YER169W | RPH1 | YLR304C | ACO1 | Glucose |  | YPL038W | MET31 | YMR189W | GCV2 | Galactose |
| YER169W | RPH1 | YLR328W | NMA1 | Glucose |  | YPL038W | MET31 | YMR205C | PFK2 | Galactose |
| YER169W | RPH1 | YLR354C | TAL1 | Glucose |  | YPL038W | MET31 | YNL277W | MET2 | Galactose |
| YER169W | RPH1 | YLR355C | ILV5 | Glucose |  | YPL038W | MET31 | YNR050C | LYS9 | Galactose |
| YER169W | RPH1 | YLR359W | ADE13 | Glucose |  | YPL038W | MET31 | YOL064C | MET22 | Galactose |
| YER169W | RPH1 | YLR377C | FBP1 | Glucose |  | YPL038W | MET31 | YPL078C | ATP4 | Galactose |
| YER169W | RPH1 | YLR395C | COX8 | Glucose |  | YPL038W | MET31 | YPL271W | ATP15 | Galactose |
| YER169W | RPH1 | YLR438W | CAR2 | Glucose |  | YPL038W | MET31 | YPR167C | MET16 | Galactose |
| YER169W | RPH1 | YLR447C | VMA6 | Glucose |  | YDR043C | NRG1 | YNR041C | COQ2 | Glycerol |
| YER169W | RPH1 | YML004C | GLO1 | Glucose |  | YDR043C | NRG1 | YOL097C | WRS1 | Glycerol |
| YER169W | RPH1 | YML008C | ERG6 | Glucose |  | YDR043C | NRG1 | YOR074C | CDC21 | Glycerol |
| YER169W | RPH1 | YML035C | AMD1 | Glucose |  | YDR043C | NRG1 | YPR033C | HTS1 | Glycerol |
| YER169W | RPH1 | YML054C | CYB2 | Glucose |  | YDR096W | GIS1 | YPR062W | FCY1 | Glycerol |
| YER169W | RPH1 | YML081C-A | ATP18 | Glucose |  | YDR096W | GIS1 | YPR113W | PIS1 | Glycerol |
| YER169W | RPH1 | YML086C | ALO1 | Glucose |  | YDR216W | ADR1 | YPR128C | ANT1 | Glycerol |
| YER169W | RPH1 | YML100W | TSL1 | Glucose |  | YDR216W | ADR1 | YPR160W | GPH1 | Glycerol |
| YER169W | RPH1 | YML120C | NDI1 | Glucose |  | YER040W | GLN3 | YPL148C | PPT2 | Glycerol |
| YER169W | RPH1 | YML126C | ERG13 | Glucose |  | YER130C | YER130c | YPL214C | THI6 | Glycerol |
| YER169W | RPH1 | YMR009W | ADI1 | Glucose |  | YFR034C | PHO4 | YPR062W | FCY1 | Glycerol |
| YER169W | RPH1 | YMR015C | ERG5 | Glucose |  | YGL073W | HSF1 | YGR282C | BGL2 | Glycerol |
| YER169W | RPH1 | YMR020W | FMS1 | Glucose |  | YGL073W | HSF1 | YHR019C | DED81 | Glycerol |
| YER169W | RPH1 | YMR054W | STV1 | Glucose |  | YGL073W | HSF1 | YHR144C | DCD1 | Glycerol |
| YER169W | RPH1 | YMR105C | PGM2 | Glucose |  | YGL073W | HSF1 | YJR130C | STR2 | Glycerol |
| YER169W | RPH1 | YMR108W | ILV2 | Glucose |  | YGL073W | HSF1 | YLR017W | MEU1 | Glycerol |
| YER169W | RPH1 | YMR120C | ADE17 | Glucose |  | YGL073W | HSF1 | YML054C | CYB2 | Glycerol |
| YER169W | RPH1 | YMR145C | NDE1 | Glucose |  | YGL073W | HSF1 | YOL097C | WRS1 | Glycerol |
| YER169W | RPH1 | YMR202W | ERG2 | Glucose |  | YGL073W | HSF1 | YOL151W | GRE2 | Glycerol |
| YER169W | RPH1 | YMR205C | PFK2 | Glucose |  | YGL073W | HSF1 | YOR155C | ISN1 | Glycerol |
| YER169W | RPH1 | YMR217W | GUA1 | Glucose |  | YHR206W | SKN7 | YMR083W | ADH3 | Glycerol |
| YER169W | RPH1 | YMR220W | ERG8 | Glucose |  | YIL101C | XBP1 | YOL058W | ARG1 | Glycerol |
| YER169W | RPH1 | YMR250W | GAD1 | Glucose |  | YIL131C | FKH1 | YOL049W | GSH2 | Glycerol |
| YER169W | RPH1 | YMR256C | COX7 | Glucose |  | YIL131C | FKH1 | YPL148C | PPT2 | Glycerol |
| YER169W | RPH1 | YMR261C | TPS3 | Glucose |  | YIL131C | FKH1 | YPR026W | ATH1 | Glycerol |
| YER169W | RPH1 | YMR272C | SCS7 | Glucose |  | YKL038W | RGT1 | YAL044C | GCV3 | Glycerol |
| YER169W | RPH1 | YMR278W | PGM3 | Glucose |  | YKL038W | RGT1 | YBL045C | COR1 | Glycerol |
| YER169W | RPH1 | YMR296C | LCB1 | Glucose |  | YKL038W | RGT1 | YBR068C | BAP2 | Glycerol |
| YER169W | RPH1 | YMR303C | ADH2 | Glucose |  | YKL038W | RGT1 | YBR263W | SHM1 | Glycerol |
| YER169W | RPH1 | YMR319C | FET4 | Glucose |  | YKL038W | RGT1 | YBR265W | TSC10 | Glycerol |
| YER169W | RPH1 | YNL009W | IDP3 | Glucose |  | YKL038W | RGT1 | YCL025C | AGP1 | Glycerol |
| YER169W | RPH1 | YNL037C | IDH1 | Glucose |  | YKL038W | RGT1 | YCR010C | ADY2 | Glycerol |
| YER169W | RPH1 | YNL071W | LAT1 | Glucose |  | YKL038W | RGT1 | YCR098C | GIT1 | Glycerol |
| YER169W | RPH1 | YNL073W | MSK1 | Glucose |  | YKL038W | RGT1 | YDL103C | QRI1 | Glycerol |
| YER169W | RPH1 | YNL129W | NRK1 | Glucose |  | YKL038W | RGT1 | YDL174C | DLD1 | Glycerol |
| YER169W | RPH1 | YNL141W | AAH1 | Glucose |  | YKL038W | RGT1 | YDL178W | DLD2 | Glycerol |
| YER169W | RPH1 | YNL169C | PSD1 | Glucose |  | YKL038W | RGT1 | YDL210W | UGA4 | Glycerol |
| YER169W | RPH1 | YNL202W | SPS19 | Glucose |  | YKL038W | RGT1 | YDR046C | BAP3 | Glycerol |
| YER169W | RPH1 | YNL220W | ADE12 | Glucose |  | YKL038W | RGT1 | YDR294C | DPL1 | Glycerol |
| YER169W | RPH1 | YNL241C | ZWF1 | Glucose |  | YKL038W | RGT1 | YDR297W | SUR2 | Glycerol |
| YER169W | RPH1 | YNL247W | YNL247w | Glucose |  | YKL038W | RGT1 | YDR453C | TSA2 | Glycerol |
| YER169W | RPH1 | YNL277W | MET2 | Glucose |  | YKL038W | RGT1 | YDR508C | GNP1 | Glycerol |
| YER169W | RPH1 | YNL280C | ERG24 | Glucose |  | YKL038W | RGT1 | YDR529C | QCR7 | Glycerol |
| YER169W | RPH1 | YNL316C | PHA2 | Glucose |  | YKL038W | RGT1 | YEL024W | RIP1 | Glycerol |
| YER169W | RPH1 | YNR016C | ACC1 | Glucose |  | YKL038W | RGT1 | YEL046C | GLY1 | Glycerol |
| YER169W | RPH1 | YNR019W | ARE2 | Glucose |  | YKL038W | RGT1 | YEL047C | YEL047c | Glycerol |
| YER169W | RPH1 | YNR034W | SOL1 | Glucose |  | YKL038W | RGT1 | YEL071W | DLD3 | Glycerol |
| YER169W | RPH1 | YNR043W | MVD1 | Glucose |  | YKL038W | RGT1 | YER015W | FAA2 | Glycerol |
| YER169W | RPH1 | YOL020W | TAT2 | Glucose |  | YKL038W | RGT1 | YFR033C | QCR6 | Glycerol |
| YER169W | RPH1 | YOL058W | ARG1 | Glucose |  | YKL038W | RGT1 | YGL012W | ERG4 | Glycerol |
| YER169W | RPH1 | YOL086C | ADH1 | Glucose |  | YKL038W | RGT1 | YGL184C | STR3 | Glycerol |
| YER169W | RPH1 | YOL097C | WRS1 | Glucose |  | YKL038W | RGT1 | YGL224C | SDT1 | Glycerol |
| YER169W | RPH1 | YOL126C | MDH2 | Glucose |  | YKL038W | RGT1 | YGR007W | MUQ1 | Glycerol |
| YER169W | RPH1 | YOL140W | ARG8 | Glucose |  | YKL038W | RGT1 | YGR055W | MUP1 | Glycerol |
| YER169W | RPH1 | YOR011W | AUS1 | Glucose |  | YKL038W | RGT1 | YGR170W | PSD2 | Glycerol |
| YER169W | RPH1 | YOR065W | CYT1 | Glucose |  | YKL038W | RGT1 | YGR183C | QCR9 | Glycerol |
| YER169W | RPH1 | YOR095C | RKI1 | Glucose |  | YKL038W | RGT1 | YGR202C | PCT1 | Glycerol |
| YER169W | RPH1 | YOR120W | GCY1 | Glucose |  | YKL038W | RGT1 | YHL032C | GUT1 | Glycerol |
| YER169W | RPH1 | YOR125C | CAT5 | Glucose |  | YKL038W | RGT1 | YHL036W | MUP3 | Glycerol |
| YER169W | RPH1 | YOR136W | IDH2 | Glucose |  | YKL038W | RGT1 | YHR001W-A | QCR10 | Glycerol |
| YER169W | RPH1 | YOR142W | LSC1 | Glucose |  | YKL038W | RGT1 | YHR037W | PUT2 | Glycerol |
| YER169W | RPH1 | YOR180C | DCI1 | Glucose |  | YKL038W | RGT1 | YIL099W | SGA1 | Glycerol |
| YER169W | RPH1 | YOR273C | TPO4 | Glucose |  | YKL038W | RGT1 | YIL155C | GUT2 | Glycerol |
| YER169W | RPH1 | YOR332W | VMA4 | Glucose |  | YKL038W | RGT1 | YJL166W | QCR8 | Glycerol |
| YER169W | RPH1 | YOR347C | PYK2 | Glucose |  | YKL038W | RGT1 | YJR001W | AVT1 | Glycerol |
| YER169W | RPH1 | YOR348C | PUT4 | Glucose |  | YKL038W | RGT1 | YJR048W | CYC1 | Glycerol |
| YER169W | RPH1 | YOR360C | PDE2 | Glucose |  | YKL038W | RGT1 | YJR057W | CDC8 | Glycerol |
| YER169W | RPH1 | YOR374W | ALD4 | Glucose |  | YKL038W | RGT1 | YJR130C | STR2 | Glycerol |
| YER169W | RPH1 | YPL036W | PMA2 | Glucose |  | YKL038W | RGT1 | YKR031C | SPO14 | Glycerol |
| YER169W | RPH1 | YPL057C | SUR1 | Glucose |  | YKL038W | RGT1 | YKR039W | GAP1 | Glycerol |
| YER169W | RPH1 | YPL061W | ALD6 | Glucose |  | YKL038W | RGT1 | YLR133W | CKI1 | Glycerol |
| YER169W | RPH1 | YPL087W | YDC1 | Glucose |  | YKL038W | RGT1 | YLR260W | LCB5 | Glycerol |
| YER169W | RPH1 | YPL231W | FAS2 | Glucose |  | YKL038W | RGT1 | YLR284C | ECI1 | Glycerol |
| YER169W | RPH1 | YPL262W | FUM1 | Glucose |  | YKL038W | RGT1 | YLR377C | FBP1 | Glycerol |
| YER169W | RPH1 | YPL265W | DIP5 | Glucose |  | YKL038W | RGT1 | YLR438W | CAR2 | Glycerol |
| YER169W | RPH1 | YPL271W | ATP15 | Glucose |  | YKL038W | RGT1 | YML082W | YML082w | Glycerol |
| YER169W | RPH1 | YPR021C | AGC1 | Glucose |  | YKL038W | RGT1 | YNL202W | SPS19 | Glycerol |
| YER169W | RPH1 | YPR035W | GLN1 | Glucose |  | YKL038W | RGT1 | YNR012W | URK1 | Glycerol |
| YER169W | RPH1 | YPR036W | VMA13 | Glucose |  | YKL038W | RGT1 | YOR171C | LCB4 | Glycerol |
| YER169W | RPH1 | YPR074C | TKL1 | Glucose |  | YKL038W | RGT1 | YOR348C | PUT4 | Glycerol |
| YER169W | RPH1 | YPR113W | PIS1 | Glucose |  | YKL038W | RGT1 | YPL111W | CAR1 | Glycerol |
| YER169W | RPH1 | YPR156C | TPO3 | Glucose |  | YKL038W | RGT1 | YPL214C | THI6 | Glycerol |
| YER169W | RPH1 | YPR159W | KRE6 | Glucose |  | YLR014C | PPR1 | YOR155C | ISN1 | Glycerol |
| YER169W | RPH1 | YPR192W | AQY1 | Glucose |  | YLR403W | SFP1 | YNL247W | YNL247w | Glycerol |
| YFR034C | PHO4 | YPR026W | ATH1 | Glucose |  | YLR403W | SFP1 | YOR126C | IAH1 | Glycerol |
| YGL073W | HSF1 | YDR058C | TGL2 | Glucose |  | YLR403W | SFP1 | YPL111W | CAR1 | Glycerol |
| YGL073W | HSF1 | YDR368W | YPR1 | Glucose |  | YML007W | YAP1 | YDR272W | GLO2 | Glycerol |
| YGL073W | HSF1 | YGR088W | CTT1 | Glucose |  | YML007W | YAP1 | YGL224C | SDT1 | Glycerol |
| YGL073W | HSF1 | YJL088W | ARG3 | Glucose |  | YML007W | YAP1 | YGR019W | UGA1 | Glycerol |
| YGL073W | HSF1 | YLL018C | DPS1 | Glucose |  | YML007W | YAP1 | YGR185C | TYS1 | Glycerol |
| YGL073W | HSF1 | YMR313C | TGL3 | Glucose |  | YML007W | YAP1 | YJR057W | CDC8 | Glycerol |
| YGL073W | HSF1 | YNR012W | URK1 | Glucose |  | YML007W | YAP1 | YML004C | GLO1 | Glycerol |
| YHR206W | SKN7 | YNL241C | ZWF1 | Glucose |  | YML007W | YAP1 | YMR250W | GAD1 | Glycerol |
| YIL131C | FKH1 | YOR209C | NPT1 | Glucose |  | YML007W | YAP1 | YNL003C | PET8 | Glycerol |
| YIL131C | FKH1 | YPL111W | CAR1 | Glucose |  | YML007W | YAP1 | YNL009W | IDP3 | Glycerol |
| YIL131C | FKH1 | YPL214C | THI6 | Glucose |  | YML007W | YAP1 | YNL202W | SPS19 | Glycerol |
| YIL131C | FKH1 | YPR062W | FCY1 | Glucose |  | YML007W | YAP1 | YNL247W | YNL247w | Glycerol |
| YJL110C | GZF3 | YOL066C | RIB2 | Glucose |  | YML007W | YAP1 | YPL160W | CDC60 | Glycerol |
| YKR099W | BAS1 | YOL143C | RIB4 | Glucose |  | YNL027W | CRZ1 | YPR006C | ICL2 | Glycerol |
| YKR099W | BAS1 | YOR202W | HIS3 | Glucose |  | YNL068C | FKH2 | YNR012W | URK1 | Glycerol |
| YLR014C | PPR1 | YPL148C | PPT2 | Glucose |  | YNL068C | FKH2 | YOR209C | NPT1 | Glycerol |
| YLR403W | SFP1 | YNL141W | AAH1 | Glucose |  | YNL068C | FKH2 | YPR183W | DPM1 | Glycerol |
| YLR403W | SFP1 | YOL143C | RIB4 | Glucose |  | YOR113W | AZF1 | YBR068C | BAP2 | Glycerol |
| YML007W | YAP1 | YFL045C | SEC53 | Glucose |  | YOR113W | AZF1 | YBR069C | TAT1 | Glycerol |
| YML007W | YAP1 | YIL020C | HIS6 | Glucose |  | YOR113W | AZF1 | YBR192W | RIM2 | Glycerol |
| YML007W | YAP1 | YJR051W | OSM1 | Glucose |  | YOR113W | AZF1 | YDL198C | GGC1 | Glycerol |
| YMR037C | MSN2 | YOR155C | ISN1 | Glucose |  | YOR113W | AZF1 | YDR046C | BAP3 | Glycerol |
| YMR037C | MSN2 | YPR113W | PIS1 | Glucose |  | YOR113W | AZF1 | YDR368W | YPR1 | Glycerol |
| YNL027W | CRZ1 | YOR374W | ALD4 | Glucose |  | YOR113W | AZF1 | YDR508C | GNP1 | Glycerol |
| YBL021C | HAP3 | YAL038W | CDC19 | Galactose |  | YOR113W | AZF1 | YEL046C | GLY1 | Glycerol |
| YBL021C | HAP3 | YAL062W | GDH3 | Galactose |  | YOR113W | AZF1 | YEL047C | YEL047c | Glycerol |
| YBL021C | HAP3 | YBL045C | COR1 | Galactose |  | YOR113W | AZF1 | YER070W | RNR1 | Glycerol |
| YBL021C | HAP3 | YBL099W | ATP1 | Galactose |  | YOR113W | AZF1 | YER178W | PDA1 | Glycerol |
| YBL021C | HAP3 | YBR018C | GAL7 | Galactose |  | YOR113W | AZF1 | YGL084C | GUP1 | Glycerol |
| YBL021C | HAP3 | YBR019C | GAL10 | Galactose |  | YOR113W | AZF1 | YGR121C | MEP1 | Glycerol |
| YBL021C | HAP3 | YBR029C | CDS1 | Galactose |  | YOR113W | AZF1 | YHL032C | GUT1 | Glycerol |
| YBL021C | HAP3 | YBR039W | ATP3 | Galactose |  | YOR113W | AZF1 | YHR104W | GRE3 | Glycerol |
| YBL021C | HAP3 | YBR132C | AGP2 | Galactose |  | YOR113W | AZF1 | YHR144C | DCD1 | Glycerol |
| YBL021C | HAP3 | YBR153W | RIB7 | Galactose |  | YOR113W | AZF1 | YJL026W | RNR2 | Glycerol |
| YBL021C | HAP3 | YCL018W | LEU2 | Galactose |  | YOR113W | AZF1 | YJR105W | ADO1 | Glycerol |
| YBL021C | HAP3 | YCR028C | FEN2 | Galactose |  | YOR113W | AZF1 | YKR039W | GAP1 | Glycerol |
| YBL021C | HAP3 | YDL067C | COX9 | Galactose |  | YOR113W | AZF1 | YLL043W | FPS1 | Glycerol |
| YBL021C | HAP3 | YDL171C | GLT1 | Galactose |  | YOR113W | AZF1 | YML008C | ERG6 | Glycerol |
| YBL021C | HAP3 | YDL181W | INH1 | Galactose |  | YOR113W | AZF1 | YNL104C | LEU4 | Glycerol |
| YBL021C | HAP3 | YDR007W | TRP1 | Galactose |  | YOR113W | AZF1 | YNL142W | MEP2 | Glycerol |
| YBL021C | HAP3 | YDR148C | KGD2 | Galactose |  | YOR113W | AZF1 | YNR001C | CIT1 | Glycerol |
| YBL021C | HAP3 | YDR298C | ATP5 | Galactose |  | YOR113W | AZF1 | YOL020W | TAT2 | Glycerol |
| YBL021C | HAP3 | YDR322C-A | TIM11 | Galactose |  | YOR113W | AZF1 | YOR348C | PUT4 | Glycerol |
| YBL021C | HAP3 | YDR341C | YDR341c | Galactose |  | YOR113W | AZF1 | YPL189W | GUP2 | Glycerol |
| YBL021C | HAP3 | YDR354W | TRP4 | Galactose |  | YOR113W | AZF1 | YPL265W | DIP5 | Glycerol |
| YBL021C | HAP3 | YDR377W | ATP17 | Galactose |  | YOR113W | AZF1 | YPR001W | CIT3 | Glycerol |
| YBL021C | HAP3 | YDR529C | QCR7 | Galactose |  | YOR113W | AZF1 | YPR138C | MEP3 | Glycerol |
| YBL021C | HAP3 | YEL024W | RIP1 | Galactose |  | YOR113W | AZF1 | YPR160W | GPH1 | Glycerol |
| YBL021C | HAP3 | YER003C | PMI40 | Galactose |  | YOR162C | YRR1 | YOL052C | SPE2 | Glycerol |
| YBL021C | HAP3 | YER026C | CHO1 | Galactose |  | YBL103C | RTG3 | YNR012W | URK1 | Ethanol |
| YBL021C | HAP3 | YER052C | HOM3 | Galactose |  | YBL103C | RTG3 | YPL111W | CAR1 | Ethanol |
| YBL021C | HAP3 | YER055C | HIS1 | Galactose |  | YDR043C | NRG1 | YPR160W | GPH1 | Ethanol |
| YBL021C | HAP3 | YER069W | ARG5,6 | Galactose |  | YER040W | GLN3 | YOR209C | NPT1 | Ethanol |
| YBL021C | HAP3 | YER070W | RNR1 | Galactose |  | YER040W | GLN3 | YPL214C | THI6 | Ethanol |
| YBL021C | HAP3 | YER178W | PDA1 | Galactose |  | YER130C | YER130c | YPR033C | HTS1 | Ethanol |
| YBL021C | HAP3 | YFL018C | LPD1 | Galactose |  | YFR034C | PHO4 | YPL214C | THI6 | Ethanol |
| YBL021C | HAP3 | YFL022C | FRS2 | Galactose |  | YGL073W | HSF1 | YDR178W | SDH4 | Ethanol |
| YBL021C | HAP3 | YGL001C | ERG26 | Galactose |  | YGL073W | HSF1 | YER065C | ICL1 | Ethanol |
| YBL021C | HAP3 | YGL009C | LEU1 | Galactose |  | YGL073W | HSF1 | YFL030W | AGX1 | Ethanol |
| YBL021C | HAP3 | YGL187C | COX4 | Galactose |  | YGL073W | HSF1 | YGL245W | GUS1 | Ethanol |
| YBL021C | HAP3 | YGL191W | COX13 | Galactose |  | YGL073W | HSF1 | YGR170W | PSD2 | Ethanol |
| YBL021C | HAP3 | YGL205W | POX1 | Galactose |  | YGL073W | HSF1 | YIR031C | DAL7 | Ethanol |
| YBL021C | HAP3 | YGR175C | ERG1 | Galactose |  | YGL073W | HSF1 | YJL045W | YJL045w | Ethanol |
| YBL021C | HAP3 | YGR183C | QCR9 | Galactose |  | YGL073W | HSF1 | YKL141W | SDH3 | Ethanol |
| YBL021C | HAP3 | YGR193C | PDX1 | Galactose |  | YGL073W | HSF1 | YKR097W | PCK1 | Ethanol |
| YBL021C | HAP3 | YGR240C | PFK1 | Galactose |  | YGL073W | HSF1 | YLL041C | SDH2 | Ethanol |
| YBL021C | HAP3 | YGR260W | TNA1 | Galactose |  | YGL073W | HSF1 | YLR164W | YLR164w | Ethanol |
| YBL021C | HAP3 | YHR001W-A | QCR10 | Galactose |  | YGL073W | HSF1 | YNL117W | MLS1 | Ethanol |
| YBL021C | HAP3 | YHR007C | ERG11 | Galactose |  | YHR006W | STP2 | Q0130 | OLI1 | Ethanol |
| YBL021C | HAP3 | YHR019C | DED81 | Galactose |  | YHR006W | STP2 | YBL099W | ATP1 | Ethanol |
| YBL021C | HAP3 | YHR051W | COX6 | Galactose |  | YHR006W | STP2 | YBR039W | ATP3 | Ethanol |
| YBL021C | HAP3 | YHR072W | ERG7 | Galactose |  | YHR006W | STP2 | YDL181W | INH1 | Ethanol |
| YBL021C | HAP3 | YHR190W | ERG9 | Galactose |  | YHR006W | STP2 | YDR298C | ATP5 | Ethanol |
| YBL021C | HAP3 | YIL111W | COX5b | Galactose |  | YHR006W | STP2 | YJR121W | ATP2 | Ethanol |
| YBL021C | HAP3 | YIL125W | KGD1 | Galactose |  | YHR006W | STP2 | YKL016C | ATP7 | Ethanol |
| YBL021C | HAP3 | YIL155C | GUT2 | Galactose |  | YHR006W | STP2 | YLR295C | ATP14 | Ethanol |
| YBL021C | HAP3 | YJL166W | QCR8 | Galactose |  | YHR206W | SKN7 | YIL124W | AYR1 | Ethanol |
| YBL021C | HAP3 | YJL167W | ERG20 | Galactose |  | YHR206W | SKN7 | YJR105W | ADO1 | Ethanol |
| YBL021C | HAP3 | YJL196C | ELO1 | Galactose |  | YHR206W | SKN7 | YKR097W | PCK1 | Ethanol |
| YBL021C | HAP3 | YJR048W | CYC1 | Galactose |  | YHR206W | SKN7 | YPR160W | GPH1 | Ethanol |
| YBL021C | HAP3 | YJR073C | OPI3 | Galactose |  | YIL101C | XBP1 | YNR012W | URK1 | Ethanol |
| YBL021C | HAP3 | YJR077C | MIR1 | Galactose |  | YIL101C | XBP1 | YNR033W | ABZ1 | Ethanol |
| YBL021C | HAP3 | YJR121W | ATP2 | Galactose |  | YIL101C | XBP1 | YPR160W | GPH1 | Ethanol |
| YBL021C | HAP3 | YKL016C | ATP7 | Galactose |  | YIL131C | FKH1 | YOL059W | GPD2 | Ethanol |
| YBL021C | HAP3 | YKL024C | URA6 | Galactose |  | YIL131C | FKH1 | YOR126C | IAH1 | Ethanol |
| YBL021C | HAP3 | YKL150W | MCR1 | Galactose |  | YIL131C | FKH1 | YOR168W | GLN4 | Ethanol |
| YBL021C | HAP3 | YLR038C | COX12 | Galactose |  | YIL131C | FKH1 | YPR183W | DPM1 | Ethanol |
| YBL021C | HAP3 | YLR043C | TRX1 | Galactose |  | YJL110C | GZF3 | YPL214C | THI6 | Ethanol |
| YBL021C | HAP3 | YLR109W | AHP1 | Galactose |  | YKL062W | MSN4 | YPR160W | GPH1 | Ethanol |
| YBL021C | HAP3 | YLR174W | IDP2 | Galactose |  | YLR223C | IFH1 | Q0085 | ATP6 | Ethanol |
| YBL021C | HAP3 | YLR209C | PNP1 | Galactose |  | YLR223C | IFH1 | YDR111C | ALT2 | Ethanol |
| YBL021C | HAP3 | YLR295C | ATP14 | Galactose |  | YLR223C | IFH1 | YIL020C | HIS6 | Ethanol |
| YBL021C | HAP3 | YLR304C | ACO1 | Galactose |  | YLR223C | IFH1 | YLR060W | FRS1 | Ethanol |
| YBL021C | HAP3 | YLR372W | SUR4 | Galactose |  | YLR223C | IFH1 | YOR095C | RKI1 | Ethanol |
| YBL021C | HAP3 | YLR395C | COX8 | Galactose |  | YLR223C | IFH1 | YOR184W | SER1 | Ethanol |
| YBL021C | HAP3 | YMR205C | PFK2 | Galactose |  | YLR223C | IFH1 | YOR236W | DFR1 | Ethanol |
| YBL021C | HAP3 | YMR256C | COX7 | Galactose |  | YLR223C | IFH1 | YPL117C | IDI1 | Ethanol |
| YBL021C | HAP3 | YMR272C | SCS7 | Galactose |  | YLR403W | SFP1 | YKR097W | PCK1 | Ethanol |
| YBL021C | HAP3 | YNL009W | IDP3 | Galactose |  | YLR403W | SFP1 | YNR041C | COQ2 | Ethanol |
| YBL021C | HAP3 | YNL052W | COX5a | Galactose |  | YLR403W | SFP1 | YOR136W | IDH2 | Ethanol |
| YBL021C | HAP3 | YNL071W | LAT1 | Galactose |  | YLR451W | LEU3 | Q0130 | OLI1 | Ethanol |
| YBL021C | HAP3 | YNL104C | LEU4 | Galactose |  | YLR451W | LEU3 | YBL099W | ATP1 | Ethanol |
| YBL021C | HAP3 | YNL169C | PSD1 | Galactose |  | YLR451W | LEU3 | YDL181W | INH1 | Ethanol |
| YBL021C | HAP3 | YNL220W | ADE12 | Galactose |  | YLR451W | LEU3 | YDR298C | ATP5 | Ethanol |
| YBL021C | HAP3 | YNR050C | LYS9 | Galactose |  | YML007W | YAP1 | YEL038W | UTR4 | Ethanol |
| YBL021C | HAP3 | YOL066C | RIB2 | Galactose |  | YML007W | YAP1 | YGR010W | NMA2 | Ethanol |
| YBL021C | HAP3 | YOL140W | ARG8 | Galactose |  | YML007W | YAP1 | YJL060W | BNA3 | Ethanol |
| YBL021C | HAP3 | YOR065W | CYT1 | Galactose |  | YML007W | YAP1 | YKL184W | SPE1 | Ethanol |
| YBL021C | HAP3 | YOR202W | HIS3 | Galactose |  | YML007W | YAP1 | YKR097W | PCK1 | Ethanol |
| YBL021C | HAP3 | YOR347C | PYK2 | Galactose |  | YML007W | YAP1 | YLR328W | NMA1 | Ethanol |
| YBL021C | HAP3 | YOR375C | GDH1 | Galactose |  | YML007W | YAP1 | YMR009W | ADI1 | Ethanol |
| YBL021C | HAP3 | YPL078C | ATP4 | Galactose |  | YML007W | YAP1 | YOR155C | ISN1 | Ethanol |
| YBL021C | HAP3 | YPL271W | ATP15 | Galactose |  | YML007W | YAP1 | YPR002W | PDH1 | Ethanol |
| YBL021C | HAP3 | YPR020W | ATP20 | Galactose |  | YML007W | YAP1 | YPR069C | SPE3 | Ethanol |
| YBL021C | HAP3 | YPR021C | AGC1 | Galactose |  | YNL068C | FKH2 | YPL110C | GDE1 | Ethanol |
| YBL021C | HAP3 | YPR128C | ANT1 | Galactose |  | YNL068C | FKH2 | YPL160W | CDC60 | Ethanol |
| YBL021C | HAP3 | YPR191W | QCR2 | Galactose |  | YOR113W | AZF1 | Q0085 | ATP6 | Ethanol |
| YBL103C | RTG3 | YOR120W | GCY1 | Galactose |  | YOR113W | AZF1 | YBL045C | COR1 | Ethanol |
| YDR043C | NRG1 | YOL052C | SPE2 | Galactose |  | YOR113W | AZF1 | YBL099W | ATP1 | Ethanol |
| YDR096W | GIS1 | YPR160W | GPH1 | Galactose |  | YOR113W | AZF1 | YBR035C | PDX3 | Ethanol |
| YER040W | GLN3 | YOL049W | GSH2 | Galactose |  | YOR113W | AZF1 | YCL040W | GLK1 | Ethanol |
| YER040W | GLN3 | YOR155C | ISN1 | Galactose |  | YOR113W | AZF1 | YCR028C | FEN2 | Ethanol |
| YFL044C | OTU1 | YAL022C | FUN26 | Galactose |  | YOR113W | AZF1 | YDL004W | ATP16 | Ethanol |
| YFL044C | OTU1 | YBR019C | GAL10 | Galactose |  | YOR113W | AZF1 | YDL015C | TSC13 | Ethanol |
| YFL044C | OTU1 | YGL001C | ERG26 | Galactose |  | YOR113W | AZF1 | YDL078C | MDH3 | Ethanol |
| YFL044C | OTU1 | YGL191W | COX13 | Galactose |  | YOR113W | AZF1 | YDL085W | NDE2 | Ethanol |
| YFL044C | OTU1 | YLR209C | PNP1 | Galactose |  | YOR113W | AZF1 | YDL103C | QRI1 | Ethanol |
| YFR034C | PHO4 | YPR183W | DPM1 | Galactose |  | YOR113W | AZF1 | YDR148C | KGD2 | Ethanol |
| YGL073W | HSF1 | YFL017C | GNA1 | Galactose |  | YOR113W | AZF1 | YDR322C-A | TIM11 | Ethanol |
| YGL073W | HSF1 | YGR185C | TYS1 | Galactose |  | YOR113W | AZF1 | YDR377W | ATP17 | Ethanol |
| YGL073W | HSF1 | YJL060W | BNA3 | Galactose |  | YOR113W | AZF1 | YEL038W | UTR4 | Ethanol |
| YGL073W | HSF1 | YJR078W | BNA2 | Galactose |  | YOR113W | AZF1 | YER015W | FAA2 | Ethanol |
| YGL073W | HSF1 | YKL004W | AUR1 | Galactose |  | YOR113W | AZF1 | YER065C | ICL1 | Ethanol |
| YGL073W | HSF1 | YKL211C | TRP3 | Galactose |  | YOR113W | AZF1 | YER091C | MET6 | Ethanol |
| YGL073W | HSF1 | YLL028W | TPO1 | Galactose |  | YOR113W | AZF1 | YFL030W | AGX1 | Ethanol |
| YGL073W | HSF1 | YLR056W | ERG3 | Galactose |  | YOR113W | AZF1 | YFR033C | QCR6 | Ethanol |
| YGL073W | HSF1 | YLR231C | BNA5 | Galactose |  | YOR113W | AZF1 | YFR055W | IRC7 | Ethanol |
| YGL073W | HSF1 | YML008C | ERG6 | Galactose |  | YOR113W | AZF1 | YGL205W | POX1 | Ethanol |
| YGL073W | HSF1 | YMR113W | FOL3 | Galactose |  | YOR113W | AZF1 | YGR088W | CTT1 | Ethanol |
| YGL073W | HSF1 | YMR169C | ALD3 | Galactose |  | YOR113W | AZF1 | YGR244C | LSC2 | Ethanol |
| YGL073W | HSF1 | YMR170C | ALD2 | Galactose |  | YOR113W | AZF1 | YHR123W | EPT1 | Ethanol |
| YGL073W | HSF1 | YNL169C | PSD1 | Galactose |  | YOR113W | AZF1 | YIL013C | PDR11 | Ethanol |
| YGL073W | HSF1 | YNL256W | FOL1 | Galactose |  | YOR113W | AZF1 | YIL160C | POT1 | Ethanol |
| YGL073W | HSF1 | YOR273C | TPO4 | Galactose |  | YOR113W | AZF1 | YJR121W | ATP2 | Ethanol |
| YHR206W | SKN7 | YPL148C | PPT2 | Galactose |  | YOR113W | AZF1 | YKL001C | MET14 | Ethanol |
| YIL101C | XBP1 | YOL055C | THI20 | Galactose |  | YOR113W | AZF1 | YKL016C | ATP7 | Ethanol |
| YIL101C | XBP1 | YOL059W | GPD2 | Galactose |  | YOR113W | AZF1 | YKL106W | AAT1 | Ethanol |
| YIL101C | XBP1 | YPL258C | THI21 | Galactose |  | YOR113W | AZF1 | YKL127W | PGM1 | Ethanol |
| YIL101C | XBP1 | YPR121W | THI22 | Galactose |  | YOR113W | AZF1 | YKL188C | PXA2 | Ethanol |
| YIL131C | FKH1 | YOR074C | CDC21 | Galactose |  | YOR113W | AZF1 | YKR097W | PCK1 | Ethanol |
| YKL038W | RGT1 | YBL068W | PRS4 | Galactose |  | YOR113W | AZF1 | YLR017W | MEU1 | Ethanol |
| YKL038W | RGT1 | YCR005C | CIT2 | Galactose |  | YOR113W | AZF1 | YLR089C | ALT1 | Ethanol |
| YKL038W | RGT1 | YDR178W | SDH4 | Galactose |  | YOR113W | AZF1 | YLR109W | AHP1 | Ethanol |
| YKL038W | RGT1 | YEL038W | UTR4 | Galactose |  | YOR113W | AZF1 | YLR295C | ATP14 | Ethanol |
| YKL038W | RGT1 | YER062C | HOR2 | Galactose |  | YOR113W | AZF1 | YLR303W | MET17 | Ethanol |
| YKL038W | RGT1 | YER099C | PRS2 | Galactose |  | YOR113W | AZF1 | YLR372W | SUR4 | Ethanol |
| YKL038W | RGT1 | YFR030W | MET10 | Galactose |  | YOR113W | AZF1 | YML042W | CAT2 | Ethanol |
| YKL038W | RGT1 | YGR088W | CTT1 | Galactose |  | YOR113W | AZF1 | YMR105C | PGM2 | Ethanol |
| YKL038W | RGT1 | YGR121C | MEP1 | Galactose |  | YOR113W | AZF1 | YMR145C | NDE1 | Ethanol |
| YKL038W | RGT1 | YGR240C | PFK1 | Galactose |  | YOR113W | AZF1 | YOL052C | SPE2 | Ethanol |
| YKL038W | RGT1 | YHR144C | DCD1 | Galactose |  | YOR113W | AZF1 | YOR011W | AUS1 | Ethanol |
| YKL038W | RGT1 | YIL053W | RHR2 | Galactose |  | YOR113W | AZF1 | YOR065W | CYT1 | Ethanol |
| YKL038W | RGT1 | YIL125W | KGD1 | Galactose |  | YOR113W | AZF1 | YOR142W | LSC1 | Ethanol |
| YKL038W | RGT1 | YJL045W | YJL045w | Galactose |  | YOR113W | AZF1 | YOR184W | SER1 | Ethanol |
| YKL038W | RGT1 | YJR095W | SFC1 | Galactose |  | YOR113W | AZF1 | YPL061W | ALD6 | Ethanol |
| YKL038W | RGT1 | YJR137C | MET5 | Galactose |  | YOR113W | AZF1 | YPL271W | ATP15 | Ethanol |
| YKL038W | RGT1 | YKL141W | SDH3 | Galactose |  | YOR113W | AZF1 | YPR020W | ATP20 | Ethanol |
| YKL038W | RGT1 | YKL181W | PRS1 | Galactose |  | YOR113W | AZF1 | YPR021C | AGC1 | Ethanol |
| YKL038W | RGT1 | YLR109W | AHP1 | Galactose |  | YOR113W | AZF1 | YPR167C | MET16 | Ethanol |
| YKL038W | RGT1 | YLR164W | YLR164w | Galactose |  | YOR162C | YRR1 | YNR043W | MVD1 | Ethanol |
| YKL038W | RGT1 | YLR304C | ACO1 | Galactose |  | YOR162C | YRR1 | YPL147W | PXA1 | Ethanol |
| YKL038W | RGT1 | YMR009W | ADI1 | Galactose |  | YOR337W | TEA1 | Q0085 | ATP6 | Ethanol |
| YKL038W | RGT1 | YMR118C | YMR118c | Galactose |  | YOR337W | TEA1 | YBR132C | AGP2 | Ethanol |
| YKL038W | RGT1 | YMR169C | ALD3 | Galactose |  | YOR337W | TEA1 | YBR153W | RIB7 | Ethanol |
| YKL038W | RGT1 | YMR170C | ALD2 | Galactose |  | YOR337W | TEA1 | YCL040W | GLK1 | Ethanol |
| YKL038W | RGT1 | YMR205C | PFK2 | Galactose |  | YOR337W | TEA1 | YDL022W | GPD1 | Ethanol |
| YKL038W | RGT1 | YMR267W | PPA2 | Galactose |  | YOR337W | TEA1 | YDL181W | INH1 | Ethanol |
| YKL038W | RGT1 | YNL142W | MEP2 | Galactose |  | YOR337W | TEA1 | YDR127W | ARO1 | Ethanol |
| YKL038W | RGT1 | YNL241C | ZWF1 | Galactose |  | YOR337W | TEA1 | YGL009C | LEU1 | Ethanol |
| YKL038W | RGT1 | YOL052C | SPE2 | Galactose |  | YOR337W | TEA1 | YGR061C | ADE6 | Ethanol |
| YKL038W | RGT1 | YOL061W | PRS5 | Galactose |  | YOR337W | TEA1 | YHR051W | COX6 | Ethanol |
| YKL038W | RGT1 | YOL064C | MET22 | Galactose |  | YOR337W | TEA1 | YJL026W | RNR2 | Ethanol |
| YKL038W | RGT1 | YOR142W | LSC1 | Galactose |  | YOR337W | TEA1 | YJL088W | ARG3 | Ethanol |
| YKL038W | RGT1 | YOR184W | SER1 | Galactose |  | YOR337W | TEA1 | YJR016C | ILV3 | Ethanol |
| YKL038W | RGT1 | YPR021C | AGC1 | Galactose |  | YOR337W | TEA1 | YJR121W | ATP2 | Ethanol |
| YKL038W | RGT1 | YPR138C | MEP3 | Galactose |  | YOR337W | TEA1 | YJR148W | BAT2 | Ethanol |
| YKL038W | RGT1 | YPR160W | GPH1 | Galactose |  | YOR337W | TEA1 | YLL018C | DPS1 | Ethanol |
| YKL038W | RGT1 | YPR167C | MET16 | Galactose |  | YOR337W | TEA1 | YLR058C | SHM2 | Ethanol |
| YKL109W | HAP4 | YAL038W | CDC19 | Galactose |  | YOR337W | TEA1 | YLR174W | IDP2 | Ethanol |
| YKL109W | HAP4 | YAL054C | ACS1 | Galactose |  | YOR337W | TEA1 | YOL126C | MDH2 | Ethanol |
| YKL109W | HAP4 | YAL062W | GDH3 | Galactose |  | YOR337W | TEA1 | YOR142W | LSC1 | Ethanol |
| YKL109W | HAP4 | YAR015W | ADE1 | Galactose |  | YOR337W | TEA1 | YOR184W | SER1 | Ethanol |
| YKL109W | HAP4 | YBL015W | ACH1 | Galactose |  | YOR337W | TEA1 | YPL262W | FUM1 | Ethanol |
| YKL109W | HAP4 | YBL045C | COR1 | Galactose |  | YPL089C | RLM1 | Q0085 | ATP6 | Ethanol |
| YKL109W | HAP4 | YBL099W | ATP1 | Galactose |  | YPL089C | RLM1 | YBL099W | ATP1 | Ethanol |
| YKL109W | HAP4 | YBR018C | GAL7 | Galactose |  | YPL089C | RLM1 | YDL004W | ATP16 | Ethanol |
| YKL109W | HAP4 | YBR019C | GAL10 | Galactose |  | YPL089C | RLM1 | YDR298C | ATP5 | Ethanol |
| YKL109W | HAP4 | YBR029C | CDS1 | Galactose |  | YPL089C | RLM1 | YPR020W | ATP20 | Ethanol |
| YKL109W | HAP4 | YBR039W | ATP3 | Galactose |  | YPL248C | GAL4 | Q0130 | OLI1 | Ethanol |
| YKL109W | HAP4 | YBR132C | AGP2 | Galactose |  |  |  |  |  |  |
| YKL109W | HAP4 | YBR145W | ADH5 | Galactose |  |  |  |  |  |  |
