# Supplementary material for: Metabolic Constraint-Based Refinement of Transcriptional Regulatory Networks
Source: PLoS Comput Biol. 2013 Dec 5;9(12):e1003370. doi: 10.1371/journal.pcbi.1003370 (PMC3857774; doi:10.1371/journal.pcbi.1003370)
Supplement: Table S3 — Analysis of alternate optimal solutions. We compared networks inferred from different flux states by introducing small changes to the expected growth rate. The similarity matrix below shows the network sizes for different growth thresholds (described in the methods section) and their similarity to each other (Table S3a). We also compared refined networks using larger changes in the growth threshold used to find v2. We once again found that while the refined network sizes changed across different thresholds, they were >95% similar to each other. These results indicate that that there is a strong global optimal state for the regulatory network and by perturbing the model and the constraints we still reach very close to the global optima. In terms of network refinement, all these results suggest that there is a core set of regulatory interactions that are removed across different constraints and conditions. (Table S3b). (DOCX) [file pcbi.1003370.s013.docx]

Supplementary Table S3a and 3b:

| Network No. | 1 | 2 | 3 | 4 | 5 |
| --- | --- | --- | --- | --- | --- |
| Growth Threshold | 0.03 | 0.04 | 0.05 | 0.06 | 0.07 |
| Network size | 23932 | 22908 | 22066 | 21967 | 21573 |
| 1 | 1 | 0.9971 | 0.9963 | 0.9980 | 0.9973 |
| 2 |  | 1 | 0.9952 | 0.9954 | 0.9949 |
| 3 |  |  | 1 | 0.9980 | 0.9973 |
| 4 |  |  |  | 1 | 0.9975 |

| Network No. | 1 | 2 | 3 | 4 | 5 | 6 | 7 | 8 | 9 | 10 |
| --- | --- | --- | --- | --- | --- | --- | --- | --- | --- | --- |
| Growth Threshold | 0 | 0.01 | 0.05 | 0.1 | 0.25 | 0.33 | 0.5 | 0.75 | 0.95 | 1 |
| Network size | 31075 | 29214 | 22063 | 19423 | 17499 | 16619 | 15181 | 14496 | 12775 | 6942 |
| 1 | 1 | 1 | 1 | 1 | 1 | 1 | 1 | 1 | 1 | 1 |
| 2 |  | 1 | 1 | 1 | 1 | 1 | 1 | 1 | 0.99 | 1 |
| 3 |  |  | 1 | 1 | 1 | 1 | 1 | 0.99 | 0.99 | 0.99 |
| 4 |  |  |  | 1 | 0.99 | 0.99 | 0.99 | 0.99 | 0.99 | 0.98 |
| 5 |  |  |  |  | 1 | 0.99 | 0.99 | 0.99 | 0.99 | 0.96 |
| 6 |  |  |  |  |  | 1 | 0.99 | 0.99 | 0.99 | 0.96 |
| 7 |  |  |  |  |  |  | 1 | 0.99 | 0.99 | 0.95 |
| 8 |  |  |  |  |  |  |  | 1 | 0.99 | 0.95 |
| 9 |  |  |  |  |  |  |  |  | 1 | 0.95 |
| 10 |  |  |  |  |  |  |  |  |  | 1 |
